# Decreased long-chain acylcarnitines from insufficient β-oxidation as potential early diagnostic markers for Parkinson’s disease

**DOI:** 10.1038/s41598-017-06767-y

**Published:** 2017-08-04

**Authors:** Shinji Saiki, Taku Hatano, Motoki Fujimaki, Kei-Ichi Ishikawa, Akio Mori, Yutaka Oji, Ayami Okuzumi, Takeshi Fukuhara, Takahiro Koinuma, Yoko Imamichi, Miho Nagumo, Norihiko Furuya, Shuko Nojiri, Taku Amo, Kazuo Yamashiro, Nobutaka Hattori

**Affiliations:** 1 Department of Neurology, Juntendo University School of Medicine, Bunkyo Tokyo, 113-8421 Japan; 2 Department of Research and Therapeutics for Movement Disorders, Juntendo University School of Medicine, Bunkyo Tokyo, 113-8421 Japan; 3 Clinical Research Center, Juntendo University, Bunkyo Tokyo, 113-8421 Japan; 4 Department of Applied Chemistry, National Defense Academy, Yokosuka, Kanagawa 239-8686 Japan

## Abstract

Increasing evidence shows that metabolic abnormalities in body fluids are distinguishing features of the pathophysiology of Parkinson’s disease. However, a non-invasive approach has not been established in the earliest or pre-symptomatic phases. Here, we report comprehensive double-cohort analyses of the metabolome using capillary electrophoresis/liquid chromatography mass-spectrometry. The plasma analyses identified 18 Parkinson’s disease-specific metabolites and revealed decreased levels of seven long-chain acylcarnitines in two Parkinson’s disease cohorts (*n* = 109, 145) compared with controls (*n* = 32, 45), respectively. Furthermore, statistically significant decreases in five long-chain acylcarnitines were detected in Hoehn and Yahr stage I. Likewise, decreased levels of acylcarnitine(16:0), a decreased ratio of acylcarnitine(16:0) to fatty acid(16:0), and an increased index of carnitine palmitoyltransferase 1 were identified in Hoehn and Yahr stage I of both cohorts, suggesting of initial β-oxidation suppression. Receiver operating characteristic curves produced using 12–14 long-chain acylcarnitines provided a large area of under the curve, high specificity and moderate sensitivity for diagnosing Parkinson’s disease. Our data demonstrate that a primary decrement of mitochondrial β-oxidation and that 12–14 long-chain acylcarnitines decreases would be promising diagnostic biomarkers for Parkinson’s disease.

## Introduction

Parkinson’s disease is the second most common neurodegenerative disease. It is characterised by prodromal symptoms including dysautonomia, sleep disturbance, depression, and decreased olfactory functions, followed by motor disturbances such as resting tremor, rigidity, postural instability, and gait difficulty^1^. Objective prospective data from participants at high risk of Parkinson’s disease indicates that subtle impacts on motor function may appear at least 2–5 years before diagnosis^2^. Muscular weakness in early Parkinson’s disease was reported in 1986^3^, and a subsequent Swedish nationwide cohort of 1,300,000 found a significant reduction of maximal upper extremity voluntary muscle strength in late adolescents who were then diagnosed with Parkinson’s disease 30 years later^4^. Although moderate to vigorous exercise protects against Parkinson’s disease onset, the underlying pathogenesis in Parkinson’s disease may cause an inability to exercise even in its preclinical stages^5–7^. Considering this, there is a great need for non-invasive, objective diagnostic methods to detect and distinguish pre- or very early–stage Parkinson’s disease with precise resolution, focusing on skeletal muscular functioning. Such diagnostic biomarkers would provide more efficient therapeutic interventions at the prospective Parkinson’s disease stage, enabling the establishment of disease-modifying strategies.

To date, analyses of cerebrospinal fluid, urine, salivary gland, serum, and plasma samples have shown some Parkinson’s disease specific changes in metabolites. For instance, steroidogenesis, tryptophan metabolism, phenylalanine metabolism, polyamine metabolism, oxidative stress markers and metabolites in xanthine pathways, such as caffeine, have been reported as putative biomarkers^8–21^. In some studies, the correlation of disease severity with identified metabolic biomarkers has not been fully assessed. In addition, most of the studies did not detail whether or not Parkinson’s disease patients with cognitive impairment, diabetes mellitus, obesity, or malignancy affecting plasma/serum metabolites were included^22, 23^. Besides, these studies have been performed at relatively low resolution with nuclear magnetic resonance or mass-spectrometry using conventional sample-separation systems like gas chromatography or liquid chromatography.

To overcome these issues, we used a moderately large cohort, with a larger validation cohort, with a relatively homogeneous racial background, under strictly controlled sample collection and inclusion criteria, and utilized the advantages of capillary electrophoresis time-of-flight mass-spectrometry (CE-TOFMS) to separate metabolites based on electric charge. As metabolites have a wide variety of characteristics in molecular weight, polarity, and ionized state, samples underwent comprehensive measurements coupling CE-TOFMS and liquid chromatography time-of-flight mass-spectrometry (LC-TOFMS). This dual analysis worked in a complementary manner to detect a large number of metabolites with high resolution and high sensitivity. With this advantage in technology, it has been reported that polar and water-soluble metabolites in several types of fluids can be efficiently quantified over diverse ranges^24–26^.

## Results

### The metabolomics datasets

**Figure 1:**
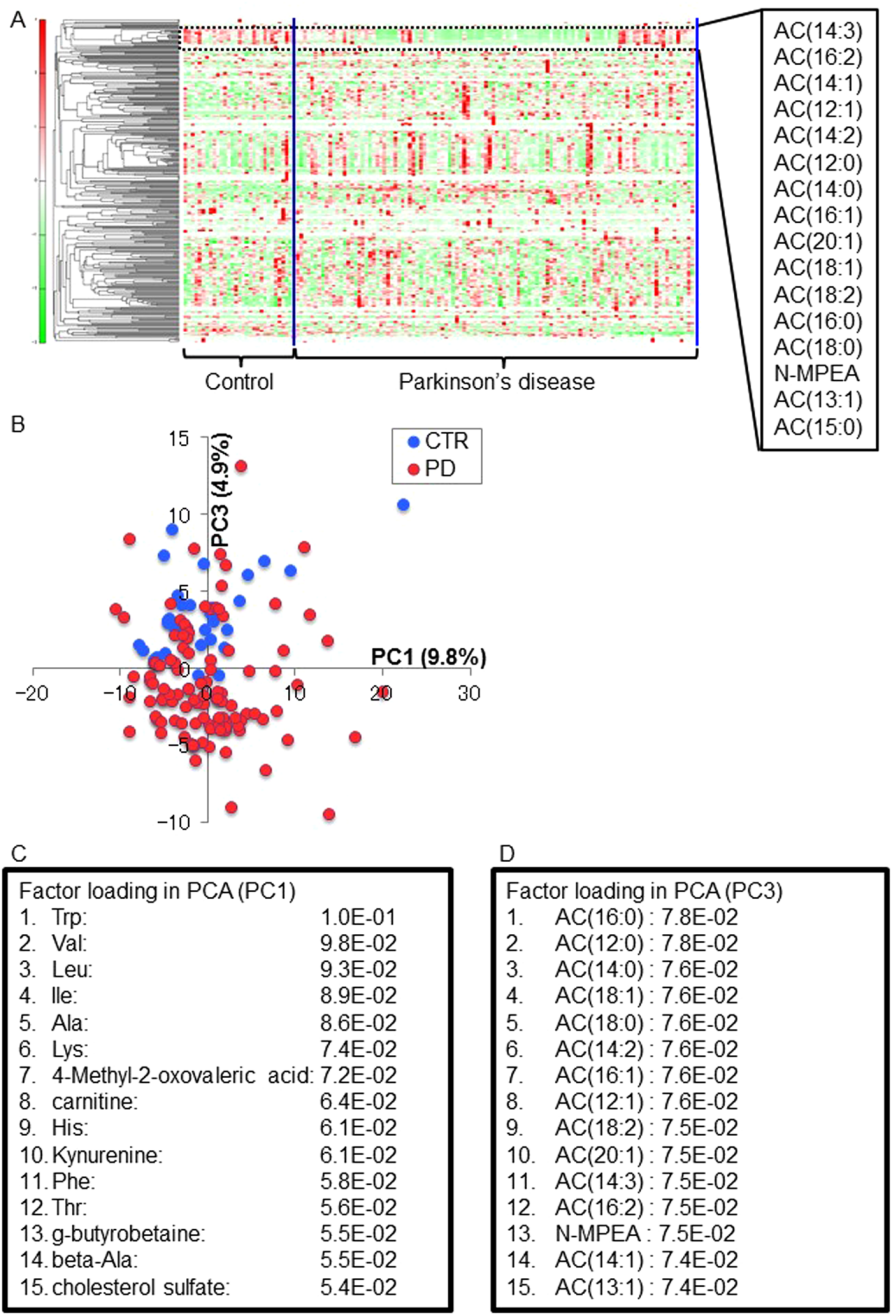
Heat map analysis and cluster analysis of observed metabolomic profiles of the 1^st^ cohort. (**A**) Red indicates higher than average metabolite concentrations, while green indicates those below average. The order of the metabolites was arranged on the basis of clustering analysis. The dotted line represents a cluster containing long-chain acylcarnitines. (**B**) Principal component analysis of participants’ plasma metabolites arranged by PC1 and PC3. (**C**,**D**) Factor loading in PC1 and PC3 listed according to statistical significance. Abbreviations: AC = acylcarnitine; CTR = control; PD = Parkinson’s disease; PC = principal component; N-MPEA = *N*-methylphenylethylamine.

The characteristics of the patients who were included in first cohort are shown in Table 1. There were no significant differences in sex (*P* = 0.473) or age (*P* = 0.0566) between controls and patients with Parkinson’s disease. The body mass index (BMI) of patients with Parkinson’s disease was significantly decreased compared with that of controls (*P* = 0.0081). We analysed the metabolomic profiles of plasma obtained from 109 patients with Parkinson’s disease and 32 controls using CE-TOFMS and LC-TOFMS. Based on their m/z values, migration times (MTs) and retention time (RTs), 124 metabolites were detected in all controls and patients with Parkinson’s disease. Metabolites detected in >50% of Parkinson’s disease patients were analyzed in detail. The metabolic data were normalized and analyzed using hierarchical cluster analysis (HCA), to provide a heat map (Fig. 1A), followed by principal component analysis (PCA) (Fig. 1B). The HCA heat map depicted the overall data structure of the study in terms of two factors involving 156 subjects and based on 318 metabolites denoted by their m/z, MT or RT, which were measured consistently in all plasma samples. We believe that these metabolomics investigations should not be based on one single cohort so we set another independent cohort (referred to as second cohort) as a validation cohort (Table 1). In this cohort metabolomics and simultaneous blood chemistries including creatine kinase, aldolase, hemoglobin A1c (HbA1c), and non-esterified fatty acid (NEFA) for confirmation of skeletal muscle metabolism were assessed. There were no significant differences between controls and those at Hoehn and Yahr (H&Y) stage I or II, in age (H&Y I: *P* = 1.00, H&Y II: *P* = 0.372) or BMI (H&Y I: *P* = 0.243, H&Y II: *P* = 0.275) (Supplementary Table S1). Eighteen Parkinson’s disease-specific metabolites [acylcarnitine isomers (12:1)-1 and (12:1)-2 are counted as one] were common to both cohorts (Table 2).

**Table 1:** Participants’ characteristics.

	1^st^ cohort			2^nd^ cohort		
	Control	Parkinson’s disease	*P*-value^a^	Control	Parkinson’s disease	*P*-value^a^
Number	32	109	—	45	145	—
Gender (Male:Female)	14:18	59:50	0.473^b^	23:22	70:75	0.740^b^
Age [years], Mean (SD)	62.9 (12.4)	67.3 (9.99)	0.0566	63.8 (15.3)	67.5 (10.2)	0.392
Duration [years], Mean (SD)	—	6.48 (5.64)	—	—	7.04 (5.61)	—
H&Y stage, (each case number)	—	I (26), II (52), III (21), IV (9), V (1)	—	—	I (41), II (60), III (35), IV (8), V (1)	—
H&Y stage, Mean (SD)	—	2.15 (0.91)	—	—	2.09 (0.897)	—
UPDRS III, Mean (SD)	—	13.9 (10.5)	—	—	14.8 (9.84)	—
MMSE, Mean (SD)	—	28.5 (1.74)	—	28.9 (2.09)	27.8 (3.14)	<0.0001
BMI [kg/m^2^], Mean (SD)	24.1 (3.88)	21.9 (2.97)	0.0081	23.2 (3.59)	22.4 (3.29)	0.0867
CK [U/ml], Mean (SD)	—	—	—	118 (47.8)	134 (91.7)	0.750
Aldolase [IU/l], Mean (SD)	—	—	—	3.64 (1.54)	3.98 (1.25)	0.0142
HbA1c [%], Mean (SD)	—	—	—	5.70 (0.378)	5.74 (0.396)	0.570
NEFA [mEq/l], Mean (SD)	—	—	—	571 (200)	613 (257)	0.420

Abbreviations: SD = standard deviation; H&Y stage = Hoehn and Yahr stage; UPDRS = Unified Parkinson’s Disease Rating Scale; MMSE = Mini Mental State Examination; BMI = Body Mass Index; CK = creatine kinase; ALD = aldolase; HbA1c = hemoglobin A1c; NEFA = non-esterified fatty acid.

^a^ *P*-value obtained by analysis of covariance between controls and PD.

^b^ *P*-value obtained by chi-squared test.

**Table 2:** Statistically significant metabolites in Parkinson’s disease vs controls in both cohorts.

Compound	Comparative Analysis			
	Parkinson’s disease/Control			
	1^st^ Cohort		2^nd^ Cohort	
	Ratio	*P*-value^a^	Ratio	*P*-value^a^
3-Methoxytyrosine	232	<*0.0001*	140	<*0.0001*
Indole-3-acetic acid	0.675	<*0.0001*	0.626	*<0.0001*
Urea	1.16	<*0.0001*	1.22	<*0.0001*
Homovanillic acid	1.34	<*0.0001*	1.28	<*0.0001*
Guanidinosuccinic acid	1.41	*0.00204*	1.32	*0.0013*
Cortisone	1.12	*0.0387*	1.50	<*0.0001*
Trigonelline	0.728	<*0.0001*	0.657	*0.0023*
Oleoylethanolamine	1.21	*0.00431*	1.13	*0.0129*
Palmitoylethanolamine	1.15	*0.00175*	1.09	*0.0168*
Citric acid	1.17	*0.00187*	1.07	*0.0252*
Deoxycholic acid	1.83	*0.0402*	2.60	<*0.0001*
AC(12:0)	0.505	<*0.0001*	0.675	*0.0146*
AC(12:1)	0.546	<*0.0001*	—	—
AC(12:1)-1	—	—	0.762	*0.0176*
AC(12:1)-2	—	—	0.632	*0.0003*
AC(14:0)	0.532	<*0.0001*	0.766	*0.0252*
AC(14:1)	0.662	*0.0043*	0.72	*0.0173*
AC(14:2)	0.628	<*0.0001*	0.735	*0.0187*
AC(16:0), Palmitoylcarnitine	0.543	<*0.0001*	0.767	*0.0205*
AC(16:1)	0.582	<*0.0001*	0.824	*0.0421*

Abbreviations: AC = acylcarnitine.

^a^ *P*-value obtained by Wilcoxon’s test, comparing between PD and controls.

### Long-chain acylcarnitines

#### Parkinson’s disease specificity

HCA is a powerful tool for deriving the associations between disease and metabolites. In the first cohort it revealed that long-chain acylcarnitines (LCACs), derived primarily from the entry of fatty acids into β-oxidation, were suppressed in Parkinson’s disease, but not in controls (Fig. 1A). PCA, is routinely used to visualize high-dimensional metabolomics data in a two- or three-dimensional subspace and can be used to identify the spectral signals responsible for the grouping or separation among the samples. It revealed that the third principal component (PC3), but not first one (PC1), effectively discriminated Parkinson’s disease cases from controls (Fig. 1B–D). To exclude the possible bias of the PCA derived from unequal sampling (32 controls and 109 Parkinson’s disease), PCA was repeatedly performed five times among 32 controls and 32 patients with Parkinson’s disease randomly selected from the first cohort; this showed similar trends to the data for the whole of the first cohort (Supplementary Fig. S1A–J). This analysis also resulted in the identification of 14 LCACs from 15 factor-loading metabolites responsible for the score plot (Fig. 1D). As summarized in Table 3, most of the short- to medium-chain ACs [AC(2:0): *P* = 0.444 (1^st^), *P* = 0.198 (2^nd^); AC(4:0): *P* = 0.0354 (1^st^), *P* = 0.109; AC(8:0): *P* = 0.645 (1^st^), *P* = 0.312 (2^nd^)]were not significantly different compared with those in controls. Seven LCACs were significantly suppressed in Parkinson’s disease compared with those in controls in both cohorts [AC(12:0): *P* < 0.0001 (1^st^), *P* = 0.0146 (2^nd^); AC(12:1): *P* < 0.0001 (1^st^); AC(12:1)-1: *P* = 0.0176 (2^nd^); AC(12:1)-2: *P* = 0.0003 (2^nd^); AC(14:0): *P* < 0.0001 (1^st^), *P* = 0.0252 (2^nd^); AC(14:1): *P* = 0.0043 (1^st^), *P* = 0.0173 (2^nd^); AC(14:2): *P* < 0.0001 (1^st^), *P* = 0.0187 (2^nd^); AC(16:0): *P* < 0.0001 (1^st^), *P* = 0.0205 (2^nd^); AC(16:1): *P* < 0.0001 (1^st^), *P* = 0.0421 (2^nd^)].

**Table 3:** List of carnitine and acylcarnitines detected in both cohorts.

Compound	Comparative Analysis			
	Parkinson’s disease/Control			
	1^st^ cohort		2^nd^ cohort	
	Ratio	*P*-value^a^	Ratio	*P*-value^a^
Creatinine	1.08	*0.0876*	0.894	0.0073
Creatine	1.01	*0.758*	0.943	*0.143*
3-Methylhistidine	0.991	*0.718*	0.998	*0.816*
Carnitine	1.02	*0.635*	0.949	0.0491
AC(2:0)	1.04	*0.444*	0.94	*0.198*
AC(4:0), Isobutyrylcarnitine	1.25	*0.0354*	0.934	*0.109*
AC(4:0), Butyrylcarnitine	—	—	1.15	*0.235*
AC(8:0)	1.09	*0.645*	0.956	*0.312*
AC(12:0)	0.505	<*0.0001*	0.675	*0.0146*
AC(12:1)	0.546	<*0.0001*	—	—
AC(12:1)-1	—	—	0.762	*0.0176*
AC(12:1)-2	—	—	0.632	*0.0002*
AC(13:1)	0.608	<*0.0001*	—	—
AC(14:0)	0.532	<*0.0001*	0.766	*0.0252*
AC(14:1)	0.662	*0.0043*	0.72	*0.0173*
AC(14:2)	0.628	<*0.0001*	0.735	*0.0187*
AC(14:3)	0.86	*0.0415*	—	—
AC(15:0)	1.02	*0.449*	—	—
AC(15:0)-1	—	—	0.292	<*0.0001*
AC(15:0)-2	—	—	0.235	<*0.0001*
AC(16:0)	0.543	<*0.0001*	0.767	*0.0205*
AC(16:1)	0.582	<*0.0001*	0.824	*0.0421*
AC(16:2)	0.716	*0.0006*	—	—
AC(18:0)	0.599	<*0.0001*	0.82	*0.0734*
AC(18:1)	0.566	<*0.0001*	0.803	*0.0739*
AC(18:2)	0.588	<*0.0001*	—	—
AC(20:1)	0.697	*0.0007*	0.952	*0.664*

Abbreviations: AC = acylcarnitine.

^a^ *P*-value obtained by Wilcoxon’s test, comparing between PD and controls.

#### Association of disease severity with LCACs

**Figure 2:**
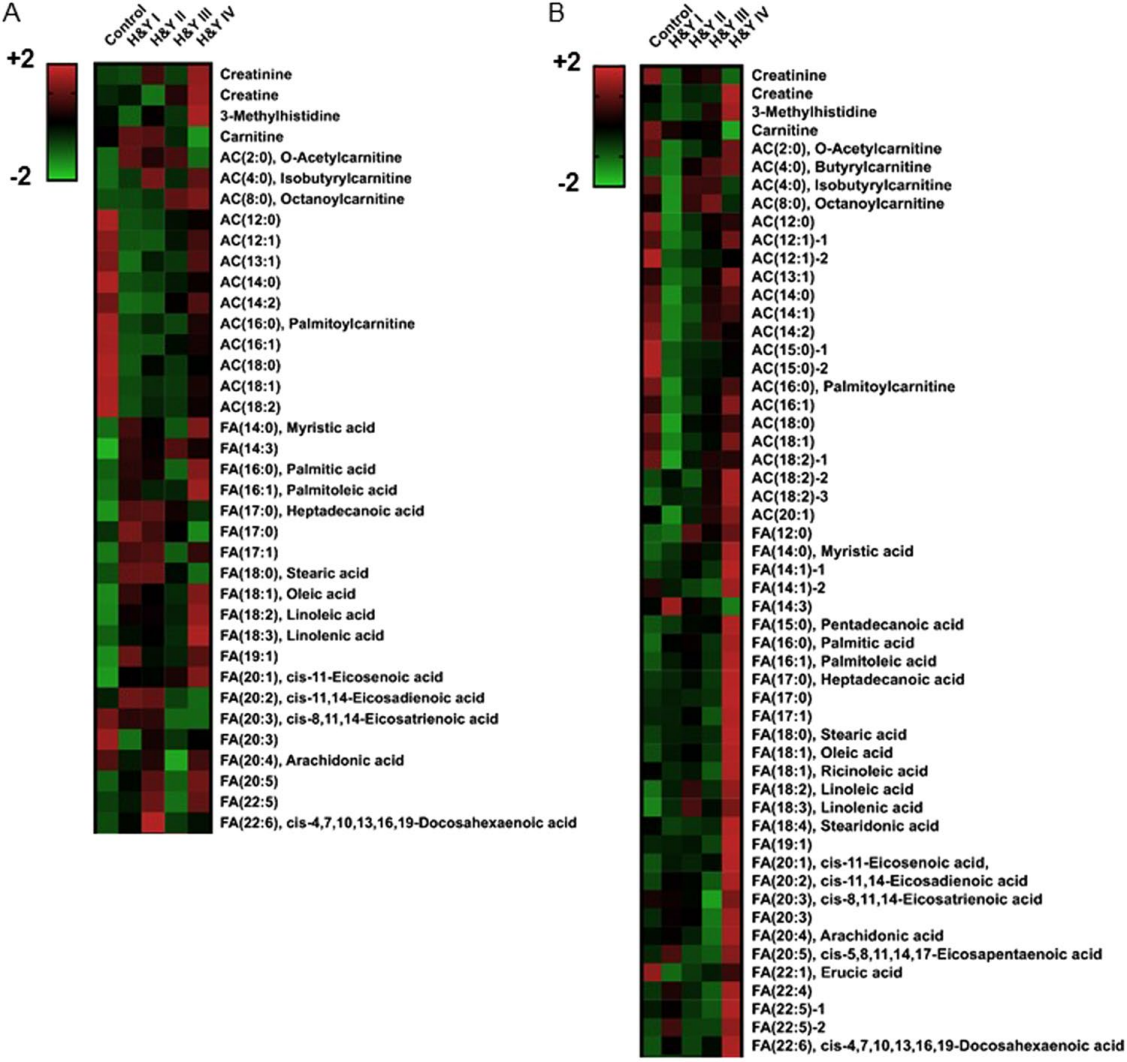
Decreased levels of long-chain acylcarnitines were detected maximally in the early stage (H&Y stage I) of Parkinson’s disease (**A**,**B**). Levels of muscular metabolites, long-chain acylcarnitines, and long-chain FAs of each Parkinson’s disease H&Y stage in each cohort are summarized in each heat map. Abbreviations: AC = acylcarnitine; H&Y = Hoehn and Yahr stage; FA = fatty acid.

To further evaluate LCACs alterations in Parkinson’s disease, we investigated the association of H&Y stages with levels of all detected LCACs. Surprisingly, the levels of five LCACs [AC(12:0): *P* < 0.0001 (1^st^), *P* = 0.0092 (2^nd^); AC(14:0): *P* < 0.0001 (1^st^), *P* = 0.0189 (2^nd^); AC(14:2): *P* = 0.0001 (1^st^), *P* = 0.0136 (2^nd^); AC(16:0): *P* < 0.0001 (1^st^), *P* = 0.0310 (2^nd^); AC(16:1): *P* = 0.0002 (1^st^), *P* = 0.0165 (2^nd^)] were significantly lower in H&Y stage I in both cohorts (Fig. 2, Supplementary Table S2). The levels of these five LCACs were slightly, but not significantly, increased in H&Y stage IV compared with those in H&Y stage I (Fig. 2, Supplementary Table S2). Most of other LCACs were also at their lowest levels in H&Y stage I. The mean age of controls (1^st^ cohort: 62.9 ± 12.4 years, 2^nd^ cohort: 63.8 ± 15.3 years) was similar to that of Parkinson’s disease patients (1^st^ cohort: 63.1 ± 10.4 years, *P* = 1.00; 2^nd^ cohort: 63.6 ± 10.4 years, *P* = 0.991) in H&Y stage I in both cohorts (Supplementary Table S1). As shown in Supplementary Table S1, mean age at sampling was generally correlated with the H&Y stage. Thus, we examined the effect of age on each level of seven LCACs that were significantly suppressed in both cohorts. Because a correlation of AC(16:1) with age was detected in both cohorts [*P* = 0.00570 (1^st^), *P* = 0.00120 (2^nd^); Supplementary Table S3], multiple analysis of variance (MANOVA) was performed to exclude the influence of disease severity (H&Y stage and UPDRS) for accurate assessment of the relationship between age and each LCAC. Only AC(16:1) positively correlated with age [H&Y: *P* = 0.126 (1^st^), *P* = 0.0263 (2^nd^); UPDRS-III: *P* = 0.00220 (1^st^), *P* = 0.00440 (2^nd^); Supplementary Table S4]. Although the BMI of Parkinson’s disease cases was decreased compared with that of controls in both cohorts (Table 1), no significant correlation of BMI with each LCAC level was detected (Supplementary Table S3).

To assess disease severity more accurately, we examined the correlation between LCACs and Unified Parkinson’s Disease Rating Scale motor section (UPDRS-III) score, which is the most widely used instrument for measuring the severity of parkinsonian motor symptoms^27^. The analysis showed no correlation between disease severity assessed by UPDRS-III and the eight LCAC levels (Supplementary Table S5).

#### Association of medication with LCACs

Treatment with levodopa effectively relieves the motor symptoms in Parkinson’s disease cases and this drug might be distributed to skeletal muscle and affect its metabolism^28, 29^. Therefore, we evaluated the effect of its medication dose and levodopa equivalent dose (LED) on the levels of LCACs by correlation analyses. As shown in Supplementary Table S6, the opposite correlation direction between the cohorts was detected, suggesting that levodopa would not modulate skeletal muscle β-oxidation. They revealed no correlation between the seven LCACs and LED (Supplementary Table S6). Based on the regimen of anti-parkinsonian mediation at sample collection, we classified Parkinson’s disease patients into those with *de novo* Parkinson’s disease (*n* = 8) and Parkinson’s disease with medication (n = 137) (Supplementary Table S7). There were no significant differences between controls and *de novo* Parkinson’s disease cases in terms of sex (*P* = 0.942), age (*P* = 0.996), Mini Mental State Examination (MMSE) score (*P* = 0.877), or BMI (*P* = 0.980). Also, the levels of most LCACs were decreased in *de novo* Parkinson’s disease compared with those in controls, with the levels of AC(12:0) (*P* = 0.0366), AC(12:1)-2 (*P* = 0.011) and AC(14:0) (*P* = 0.0366) being significantly reduced (Supplementary Table S8). Taken together, the decrease of LCACs in early Parkinson’s disease appear not to be a secondary phenomenon due to anti-parkinsonian medication, but a primary change related to the disease process.

#### Association of motor fluctuations with LCACs

Changes of skeletal muscle tone due to the wearing off phenomenon and/or dyskinesia (defined here as motor fluctuations) in Parkinson’s disease might influence muscle metabolism, including β-oxidation. As shown in Supplementary Table S9, the levels of two LCACs were significantly elevated in the second cohort, whilst the opposite trend was detected in the first cohort. Next, we investigated the effects of motor fluctuations on β-oxidation in each H&Y stage. Mildly elevated LCAC ratios of Parkinson’s disease patients with motor fluctuations compared with those in patients without such fluctuations were detected in H&Y I of both cohorts and H&Y III of the second cohort (Supplementary Table S10). Likewise, each ratio of LCAC of H&Y II-IV to H&Y I did not show a mutual direction between the two cohorts (Supplementary Fig. S2). Considering the result that the largest decrease of levels of LCACs was detected in H&Y I in both cohorts, we concluded that no apparent one-directional effects on skeletal muscle β-oxidation were induced by motor fluctuations.

#### Diagnostic values of LCACs for Parkinson’s disease or Parkinson’s disease (H&Y I) compared with controls

**Figure 3:**
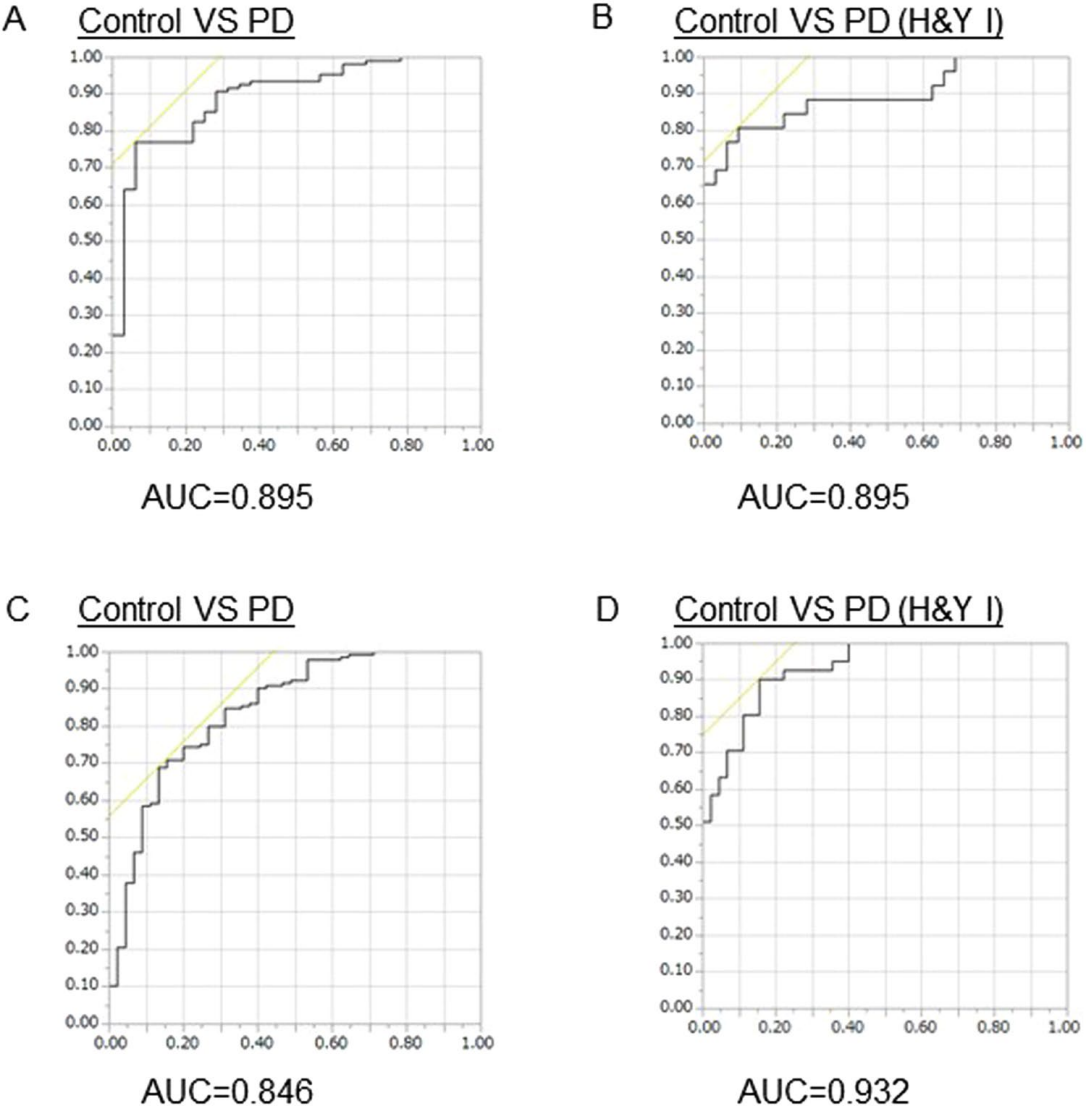
Diagnostic values of combined long-chain acylcarnitines. (**A**,**B**) Receiver operating characteristics (ROC) curves (solid) for plasma LCACs and corresponding AUC statistics for the true positive rate for Parkinson’s disease diagnosis in the 1^st^ cohort. A is for Parkinson’s disease, and B for H&Y stage I Parkinson’s disease. (**C**,**D**) ROC curves (solid) for plasma LCACs and corresponding AUC statistics for the true positive rate for Parkinson’s disease diagnosis in the 2^nd^ cohort. C is for Parkinson’s disease and D for H&Y stage I Parkinson’s disease. Abbreviations: Parkinson’s disease = PD; H&Y = Hoehn and Yahr stage; AUC = area under the curve.

As discussed, the decrease of LCACs was specific to Parkinson’s disease, especially in the early stages of the disease. Therefore, we next examined the diagnostic power of these metabolites. We selected LCACs detected in >50% of Parkinson’s disease cases in both cohorts [1^st^ cohort: AC(12:0), (12:1), (13:1), (14:0), (14:1), (14:2), (15:0), (16:0), (16:1), (18:0), (18:1), (20:1); 2^nd^ cohort: AC(12:0), (12:1)-1, (12:1)-2, (13:1), (14:0), (14:1), (14:2), (15:0)-1, (15:0)-2, (16:0), (16:1), (18:0), (18:1), (20:1)]. We detected a significant overlap of each LCAC between controls and Parkinson’s disease patients. To overcome this issue, we performed multiple logistic regression analyses to evaluate the diagnostic value of the LCACs sets and described the appropriate receiver operating characteristic (ROC) curves with area of under the curve (AUC) values in each cohort (Fig. 3, Supplementary Table S11). A set of LCACs could differentiate Parkinson’s disease H&Y I from controls with mild efficiency [AUC = 0.895 (1^st^ cohort), 0.931 (2^nd^); sensitivity = 0.808, 0.902; specificity = 0.906, 0.844; cut-off value = 0.0545, 0.0127, respectively], and was also useful for diagnosing Parkinson’s disease [AUC = 0.895 (1^st^ cohort), 0.846 (2^nd^); sensitivity = 0.771, 0.690; specificity = 0.938, 0.867; cut-off value = −1.50, −1.52)].

#### Underlying mechanisms for LCACs changes

**Figure 4:**
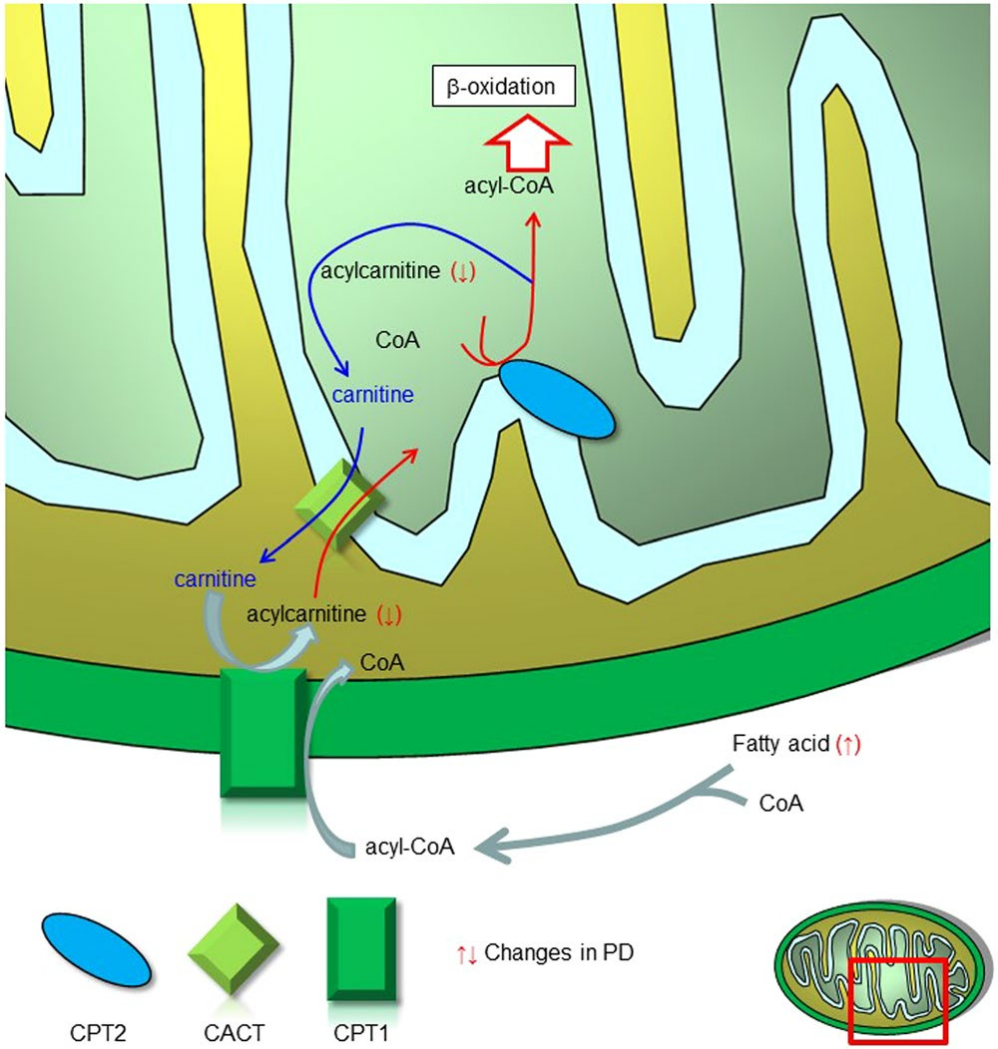
Schematic figure of carnitine shuttle in the mitochondria. Acyl-CoA, derived from fatty acid, is unable to penetrate the mitochondrial outer membrane. Using carnitine palmitoyltransferase activity, acyl-CoA is transformed to acylcarnitine, which is then shuttled into the mitochondrial matrix by carnitine-acylcarnitine translocase. Finally, acylcarnitine is converted to acyl-CoA by carnitine palmitoyltransferase 2 localized on the inner mitochondrial membrane. Abbreviations: CPT1 = carnitine palmitoyltransferase I; CPT2 = carnitine palmitoyltransferase II; CACT = carnitine-acylcarnitine translocase; PD = Parkinson’s disease.

**Figure 5:**
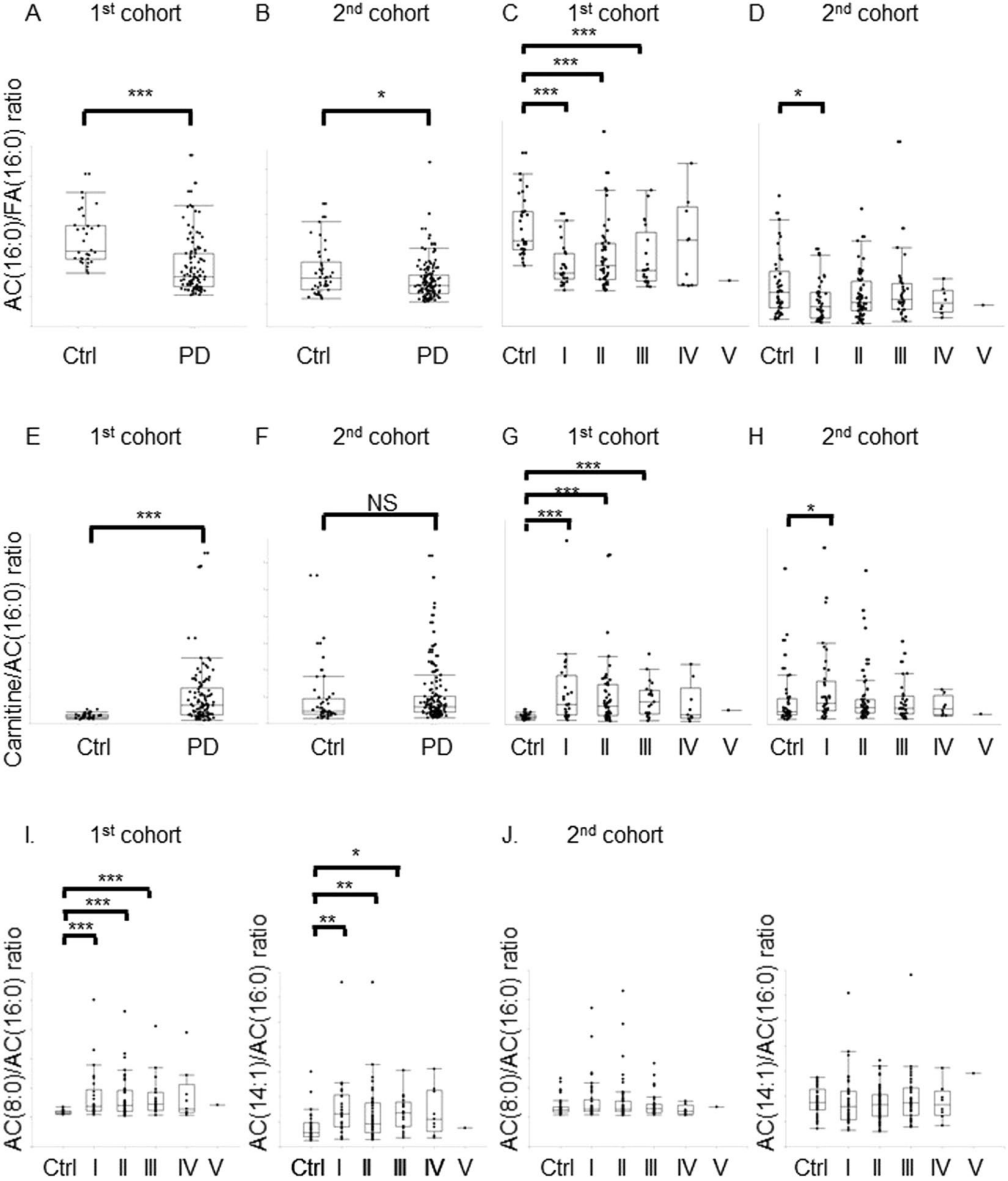
CPT1-associated indicators were significantly changed especially in early Parkinson’s disease stages. (**A**,**B**) AC(16:0)/FA(16:0) ratios of Parkinson’s disease were significantly decreased in both cohorts. *P < 0.05; ***P < 0.001 (Wilcoxon’s test). (**C**,**D**) Multiple comparisons for the ratios, AC(16:0)/FA(16:0) and carnitine/AC(16:0). The decrease of AC(16:0)/FA(16:0) ratios were mainly detected in the early stage of Parkinson's disease. *P < 0.05; ***P < 0.001 (Steel’s test). (**E**,**F**) Carnitine/AC(16:0) ratios were increased significantly in the 1^st^ cohort, but not significantly in the 2^nd^ cohort. (**G**,**H**) Carnitine/AC(16:0) ratios were significantly increased in H&Y stage I. *P < 0.05; ***P < 0.001 (Wilcoxon’s test). (**I**,**J**) Ratios of AC(8:0)/AC(16:0) and AC(14:1)/AC(16:0) in each H&Y stage. Similar increase of AC(8:0)/AC(16:0) ratio in H&Y stage I was detected, however, this was not significant in the 2^nd^ cohort. *P < 0.05; ***P < 0.001 (Steel’s test). Abbreviations: AC = acylcarnitine; FA = fatty acid; Error bars, S.D.

Similar quantities of NEFA are readily activated into acyl-coenzyme A (acyl-CoA). LCACs formed from acyl-CoA by carnitine palmitoyltransferase 1 (CPT1) in the mitochondrial outer membrane can either pass into the mitochondrial matrix via carnitine-acylcarnitine translocase or can exit the cell for disposal (Fig. 4)^30^. This CPT1-dependent regulation limits levels of AC(16:0), AC(16:1), AC(18:0) and AC(18:1), leading to an elevation in the ratio of carnitine to AC(16:0)^31^. In both cohorts, levels of AC(16:0) [*P* < 0.0001 (1^st^), *P* = 0.031(2^nd^)] and AC(16:1) [*P* = 0.0002, *P* = 0.0165 (2^nd^)] were significantly decreased in Parkinson’s disease H&Y stage I compared with those in controls (Supplementary Table S2). The ratio of AC(16:0) to FA(16:0) [*P* < 0.0001 (1^st^), *P* = 0.0167 (2^nd^)], which reflects β-oxidation input intensity, was also significantly decreased, and the ratio of carnitine to AC(16:0) [*P* < 0.0001 (1^st^), *P* = 0.044 (2^nd^)] was increased in H&Y stage I (Fig. 5A–H), indicating that CPT1-based limitation of β-oxidation flux occurs in Parkinson’s disease. Inside mitochondria, LCACs are de-esterified back to acyl-CoA and enter into the β-oxidation spiral. In this context, additive indexes, ratios of AC(8:0) to AC(16:0) [*P* < 0.0001 (1^st^), *P* = 0.0692 (2^nd^)] and AC(14:1) to AC(16:0) [*P* < 0.0001 (1^st^), *P* = 0.350 (2^nd^)]^32^, were heterogeneous in each cohort (Fig. 5I,J). Increased ratios of AC(8:0) to AC(16:0) (later-step index of β-oxidation) [*P* < 0.0001 (1^st^), *P* = 0.317 (2^nd^)]^32^ in both cohorts and preserved levels of AC(2:0) and AC(4:0) despite decreased β-oxidation input in Parkinson’s disease suggest that mitochondrial β-oxidation would be insufficient.

AC(3:0) and AC(5:0) derive from the catabolism of branched-chain amino acids (BCAAs, Leu, Ile and Val), while AC(4:0) is synthesized from both BCAAs and fatty acids (FAs). In concert with elevated AAs, concomitant increases in plasma ACs derived from amino acid oxidation occur, potentially reflecting increased amino acid flux^33, 34^. Unfortunately, neither AC(3:0) nor AC(5:0) was detected in this study. There were no significant differences in BCAA levels between the groups (Supplementary Table S12), whereas the levels of AC(2:0) [*P* = 0.444 (1^st^), *P* = 0.198 (2^nd^)], and AC(4:0) [*P* = 0.0354 (1^st^), *P* = 0.109 (2^nd^)] were mildly increased compared with the levels of LCACs in Parkinson’s disease. This implies that β-oxidation fueled by the BCAAs is not affected in Parkinson’s disease.

#### Association of skeletal muscle mass with LCACs

In the first cohort, levels of creatine (*P* = 0.758), creatinine (*P* = 0.0876), carnitine (*P* = 0.635), and 3-methylhistidine (*P* = 0.718), all of which are thought to relate to skeletal muscles mass^23^, were not significantly different in Parkinson’s disease compared with those in controls (Table 3). Their levels in each H&Y stage were also not significantly altered compared with those in (Supplementary Fig. S3). Conversely, in the second cohort, creatinine (*P* = 0.0073) and carnitine (*P* = 0.0491) levels were significantly reduced in Parkinson’s disease compared with those in controls (Table 3). In relation to H&Y stage, the levels of creatinine significantly decreased with disease severity, as determined by the Kruskal-Wallis test (*P* = 0.0093), and the levels of creatinine in H&Y stages I (*P* = 0.0061) and IV (*P* = 0.0044) were significantly decreased compared with those in the controls (Supplementary Fig. S3B). However, the levels of CK and aldolase, which increase during the muscular proteolysis phase, were not changed in any H&Y stage compared with those in controls (Supplementary Fig. S3C). Considering the gradual increase of creatine and 3-methylhistidine, as well as the decrease of carnitine, correlating with disease severity in both cohorts, slight-to-mild rhabdomyolysis with loss of muscle mass might occur in advanced Parkinson’s disease. In summary, in the early stage of Parkinson’s disease in the first cohort, apparent loss of skeletal muscle mass is unlikely, while Parkinson’s disease in H&Y stage I of the second cohort might suffer from a slight-to-mild decrease of total bodily skeletal muscle mass.

#### Association of obesity and diabetes mellitus with LCACs

Plasma and skeletal muscle concentrations of LCACs are modestly increased in individuals with insulin-resistant obesity or type 2 diabetes mellitus (T2DM)^31, 32, 35–38^. As shown in Supplementary Table 1, the BMI of those at early H&Y stages (I, II) in both cohorts was not reduced significantly compared with that in controls, implying that the early stage Parkinson’s disease patients in this study did not show excessive weight loss. Likewise, no differences in each of the LCAC concentrations [AC(12:0), AC(14:0), AC(14:1), AC(14:2), AC(16:0), AC(16:1)] were detected between Parkinson’s disease patients with T2DM and those without it. To exclude the influence of glucose tolerance on muscular metabolism, we examined the association of LCACs with disease severity in the second cohort. Kruskal-Wallis test revealed no correlation between HbA1c levels and H&Y stages (*P* = 0.241) (Supplementary Fig. S3D). According to the multivariate logistic regression analysis performed to adjust for BMI or HbA1c, neither of them significantly affected the levels of LCACs in each H&Y stage.

### Free fatty acid profiles

Fatty acids (FAs) with >22 carbons are primarily degraded in peroxisomes. Mitochondrial acyl-CoA synthase hydrolyses FAs with <22 carbons to acyl-CoAs, which are then converted to LCACs by CPT1^30^. The levels of FA(14:3) (*P* < 0.0001) and FA(20:1) (*P* = 0.0418) in Parkinson’s disease patients in the first cohort, and FA(12:0) (*P* = 0.0055), FA(14:0) (*P* = 0.0351) and FA(15:0) (*P* = 0.0294) in Parkinson’s disease patients in the second cohort were significantly increased compared with those in controls (Supplementary Table S12). Also, 18 of the 24 long-chain FAs in the first cohort, and 18 of the 26 long-chain FAs in second cohort were increased in Parkinson’s disease (Supplementary Table S12). In terms of the association of long-chain FA levels with Parkinson’s disease severity (H&Y stage), the levels of most long-chain FAs were increased even in H&Y stage I (1^st^ cohort: 18 of 25 long-chain FAs, 2^nd^ cohort: 20 of 26 long-chain FAs) (Fig. 2, Supplementary Table S13). These results suggest that the increased levels of long-chain FAs, along with decreased levels of LCACs, in early Parkinson’s disease might be associated with insufficient flux of downstream β-oxidation (Fig. 4). In the second cohort, we additionally assessed NEFA, which correspond to the total levels of long-chain FAs. The levels of NEFA in Parkinson’s disease were mildly but not significantly increased in H&Y stages I (1.06 ± 0.498, *P* = 0.999) and II (1.07 ± 0.474, *P* = 0.962) (Supplementary Table S13).

### Tyrosine metabolism

Levels of cerebrospinal fluid homovanillic acid (HVA), mainly derived from the nigrostriatal dopaminergic pathway, are reduced in Parkinson’s disease patients^39, 40^. Plasma concentrations of 3-Methoxytyrosine (*P* < 0.0001) and HVA (*P* < 0.0001) were significantly elevated in Parkinson’s disease compared with those in controls (Table 2). 3-Methoxytyrosine and HVA are dopamine pathway metabolites, so there is a possibility that these might be affected by levodopa treatment in Parkinson’s disease. Consistent with this, in both cohorts, analysis of covariance revealed that 3-methoxytyrosine [*P* = 0.0017 (1^st^); *P* < 0.0001 (2^nd^)] and HVA [*P* = 0.0187 (1^st^), *P* < 0.0001 (2^nd^)] levels correlate with LED (Supplementary Fig. 4A,B,D,E). As shown in Supplementary Table S7, there were eight *de novo* Parkinson’s disease patients in the second cohort. Levels of 3-methoxytyrosine were significantly suppressed in the *de novo* Parkinson’s disease patients or controls compared with those in Parkinson’s disease patients, suggesting that 3-methoxytyrosine is mainly derived from levodopa medication (Supplementary Fig. 4C). On the contrary, levels of HVA were suppressed in *de novo* Parkinson’s disease compared with those in controls, implying that the disruption of catecholamine metabolism occurs in early Parkinson’s disease (Supplementary Fig. 4F).

### Oleoylethanolamine and palmitoylethanolamine

*N*-Acylethanolamines are contained in endogenous molecules for anti-inflammation, as a class of lipidic mediator molecules composed of a fatty acid and ethanolamine. They include oleoylethanolamine (OEA) and palmitoylethanolamine (PEA), both of which were significantly elevated in Parkinson’s disease compared with their levels in controls in this study [OEA: *P* = 0.00431 (1^st^), *P* = 0.0129 (2^nd^); PEA: *P* = 0.00175 (1^st^), *P* = 0.0168 (2^nd^)] (Table 2). OEA activates FA β-oxidation through peroxisome proliferator-activated receptor-α, implying that OEA might be elevated due to compensatory effect against suppressed β-oxidation in early Parkinson’s disease^41^.In addition, OEA also induces autophagy^42^, a neuroprotective response to neurodegenerative diseases^43, 44^, thus it would be expected that a compensative reaction against Parkinson’s disease pathogenesis would occur.

## Discussion

In this double-cohort study, we found that patients with Parkinson’s disease had substantial reductions in seven LCACs and increased long- chain FA levels compared with controls. Additionally, the levels of five LCACs were suppressed in H&Y stage I. A decreased ratio of AC(16:0) to FA(16:0) and an increased ratio of carnitine to AC(16:0) were detected only in H&Y stage I, suggesting that early Parkinson’s disease patients suffer from β-oxidation disruption characterised by insufficient CPT1 activity. The LCAC decrement was evident in an early disease stage, as well as in *de novo* Parkinson’s disease, and not correlated with BMI, dose of levodopa, LED, or levels of HbA1c or muscle metabolites/enzymes. MANOVA revealed that, among the LCACs, only AC(16:1) was positively correlated with age in Parkinson’s disease after normalization for disease severity. The metabolomics analysis showed consistent results across cohorts, suggesting that the association of the β-oxidation disturbance with Parkinson’s disease is not likely to be due to chance. Collectively, these data have shown FA β-oxidation insufficiency is detectable, especially in early stage Parkinson’s disease, and a set of corresponding LCACs might be promising as diagnostic biomarkers enabling us to differentiate Parkinson’s disease patients from controls.

Plasma LCACs are derived from skeletal muscles and other tissues like liver and cardiac muscles^45^. In a study in which elevated LCACs were obtained from serum and skeletal muscles from insulin-resistant rats, no increase was detected in the liver, suggesting that serum LCACs originate mainly from skeletal muscles^23^. Additionally, carnitine is mainly reserved in skeletal muscles (95% of the total body store) and FA oxidation mostly occurs in the skeletal muscles, in combination with LCACs synthesis. However, the levels of four metabolites (creatinine, creatine, 3-methylhistidine, and carnitine) and BCAAs, which correlate with skeletal muscle mass^46^, were not significantly different between controls and Parkinson’s disease cases in the first cohort. On the contrary, in the second cohort, Parkinson’s disease patients had significant decreases in the levels of carnitine and creatinine, which were decreased even in H&Y stage I. Thus, we cannot entirely exclude the likelihood that mild atrophy of the skeletal muscles may already exist at this stage. Although morbid obesity causes impaired β-oxidation along with elevation of long-chain FA by several reports^33^, it remains unclear whether mild leanness would improve β-oxidation. In this study, the less statistically significant differences in the seven LCACs in the second cohort than in the first cohort may be derived from the skeletal muscle decrement.

Aerobic exercise affects skeletal muscle metabolism including β-oxidation^23^. In obese patients, static levels of LCACs are elevated due to insufficient β-oxidation, and a 10-week exercise program improves β-oxidation and lipotoxicity based on significant decreases in the levels of total carnitines and LCACs [AC(14:0) to AC(18:0)]^47^. Conversely, control subjects do not show changes in the levels of LCACs by short or long-term exercise. One study in control subjects has shown elevated levels of AC(8:0), AC(10:0), and AC(12:0) immediately after aerobic exercise, but no changes in LCACs levels were detected 3 or 24 hours after the aerobic exercise^48^. Also, a study evaluating a potential daily variation of 45 ACs have shown that concentrations of ACs rise after activity and decrease with eating^49^. In this study, blood samples were collected without exercise loading and with overnight fasting. Likewise, no correlations between LCACs and LED were detected, implying that the side effects of medications on skeletal muscles did not contribute to LCACs decrease. The findings revealed both the lowest levels of LCACs in H&Y stage I and gradually increased levels of LCACs as well as long-chain FAs in H&Y stages II-IV. Although motor fluctuations increased the levels of LCACs in H&Y I in both cohorts, similar changes were not identified in H&Y stages II-IV. In addition, no mutual trends of LCACs associated with H&Y stages and motor fluctuations were detected between the two cohorts. Based on the fact that progressed Parkinson’s disease cases probably present with levodopa-induced dyskinesia coexisting with transient relief of excess muscular stiffness by anti-parkinsonian medications, future studies should be performed to determine the accurate independent association of skeletal muscle β-oxidation with the on/off period time or dyskinesia of Parkinson’s disease quantitatively assessed by UPRDS-IV or other scales. Taking these findings together, we conclude that the LCACs decrease in at least the early stage of Parkinson’s disease in this study arises from primary changes in the skeletal muscles unrelated to exercise and medication.

The rates of FA oxidation are impaired in patients with obesity and T2DM, leading to elevation of free FAs, and short- to long-chain ACs^22, 31^. In our study, no differences in BMI between the H&Y stages were observed. Multiple variate regression models showed no significant effect of LCACs levels on BMI and HbA1c. Likewise, no significant differences in the levels of long-chain FAs and LCACs between Parkinson’s disease with or without T2DM were detected. The BMI of Parkinson’s disease patients would be expected to decrease, given the findings from an epidemiological investigation showing that patients with Parkinson’s disease have a tendency to gradually lose weight^50^. However, although the BMI of Parkinson’s disease cases was significantly lower than controls in the first cohort, it was not in the second cohort. In addition, no correlations of each LCAC with BMI were detected in both cohorts, suggesting that obesity hardly affects the levels of LCACs in Parkinson’s disease.

The potential mechanisms that underlie mitochondrial dysfunction in Parkinson’s disease are yet to be fully elucidated. In platelets, lymphocytes and skeletal muscles from Parkinson’s disease patients, respiratory chain defects are detected^51–53^. The ratio of carnitine to AC(16:0), which is increased when CPT1 activity is blocked, was markedly increased only in H&Y stage I in both cohorts. In the second cohort, the levels of carnitine were decreased in all H&Y stages, which may lead to the carnitine/AC(16:0) ratio decrease and less statistical significance in the second cohort. Likewise, the AC(16:0)/FA(16:0) ratio was significantly decreased, suggesting blocking of the entry of long-chain FAs into mitochondria for β-oxidation. Malonyl-CoA accumulation inhibits CPT1-mediated non-esterified FA into mitochondria^54^. However, we cannot exclude this possibility because malonyl-CoA was not detected in any sample in this study. Although β-oxidation input [LCACs from AC(14:0) to AC(18:2)] was significantly decreased, the levels of short- to medium-chain ACs [AC(2:0), AC(4:0) AC(8:0)] increased mildly in Parkinson’s disease. Furthermore, the latter step index of β-oxidation [AC(8:0)/AC(16:0)] was concomitantly increased with apparent (1^st^ cohort) or borderline (2^nd^ cohort) statistical significance. Apart from decreases in LCACs, most of the long-chain FAs were mildly increased in all stages of Parkinson’s disease and in *de novo* Parkinson’s disease. This is consistent with a report showing decreased β-oxidation by irreversible CPT1 inhibition by etomoxir^55^. Therefore, CPT1-based flux as well as mitochondrial β-oxidation in the whole process is thought to be suppressed primarily in the H&Y stage I of Parkinson’s disease.

Several lines of evidence have suggested that mitochondrial dysfunction, including in the respiratory chain, release of reactive oxygen species, and mitophagy are associated with a Parkinson’s disease pathogenesis^56^. Mitochondrial respiratory dysfunction has been reported in skeletal muscle biopsy specimens from Parkinson’s disease patients^57, 58^. Very recently, we identified a novel causative gene for Parkinson’s disease (*CHCHD2*) localized in the mitochondrial intermembrane space. *In silico* analyses show the association of CHCHD2 with mitochondrial respiratory chain function^59^. This study, for the first time, has revealed that FA β-oxidation, another metabolic function of mitochondria, is suppressed even in early Parkinson’s disease, consistent with prodromal skeletal muscle dysfunction even in adolescents who later go on to develop Parkinson’s disease^4^.

Metabolomic evidence of Parkinson’s disease pathogenesis and FA oxidation has been reported, especially in short- to medium-chain ACs. Using serum Parkinson’s disease samples analysed with LC-MS, AC(8:0) detected by projection to latent structure analysis can differentiate between Parkinson’s disease-phenotypes (slowly or rapidly progressing)^11^. Additionally, a study using urine samples from Parkinson’s disease patients showed elevated levels of AC(3:0) in the intermediate Parkinson’s disease stages, leading to speculation of an underling disturbance of mitochondrial FA β-oxidation^12^. Profiling of serum/plasma carnitine/LCACs from newborns by tandem MS is a well-established technique for the economical determination of the genetic defects of FA oxidation and in the electron transport chain^60^, and thus it could also be applied as an early Parkinson’s disease diagnostic tool.

Some limitations of this study should be considered. First, Parkinson’s disease diagnoses were acquired in a single university hospital. In addition, the clinical stages were assessed in the on-phase of Parkinson’s disease. If we could identify both phases (on and off), the clinical correlations of LCACs with disease severity could be assessed more accurately. The number of H&Y stage V cases in each cohort was insufficient as we had to exclude cases with other complications such as a history of aspiration pneumonia, cancer, and other inflammatory diseases. Furthermore, we need to take into consideration that AC(16:1) is mildly affected by age at sampling. Future studies should thus be performed to address whether insufficient β-oxidation of skeletal muscle is specific for patients with Parkinson’s disease or patients with parkinsonian syndromes. Also, we need to perform additional physiological research on the differentiation of β-oxidation modulation by various muscle tone changes, such as intermittent or continuous skeletal muscle movements, leading to identification of phenotypes of Parkinson’s disease, such as tremor-dominant and postural instability-gait difficulty variants^61^.

In conclusion, we identified 18 Parkinson’s disease-specific or -nonspecific, disease severity-independent diagnostic biomarkers. Of them, a set of LCACs show promise as a tool for diagnosing Parkinson’s disease due to: 1) their high specificity for Parkinson’s disease; 2) the possibility of detection in the early disease stage; and 3) the insight that they provide into disease pathogenesis (β-oxidation insufficiency). Also, by combining them with the assessment of FA(16:0), an accurate diagnosis of Parkinson’s disease could be achieved. The validity of these markers needs to be evaluated in presymptomatic Parkinson’s disease in future clinical and experimental studies.

## Materials and Methods

### Ethics statement

This study protocol complied with the Declaration of Helsinki and was approved by the ethics committee of Juntendo University. Written informed consent has been given by all participants.

### Study group

Table 1 shows the population characteristics of both cohorts. Parkinson’s disease was diagnosed according to the Movement Disorders Society Clinical Diagnostic Criteria for Parkinson’s disease^62^. We excluded Parkinson’s disease patients with dementia (less than 24 on MMSE). Neither patients nor controls had a history of tumour, cancer, aspiration pneumonia or inflammatory diseases including collagen vascular diseases. Participants suffering from acute infectious diseases or acute/chronic renal failure at the time of sample collection were also excluded.

Cohort 1 consisted of 109 patients with Parkinson’s disease, and 32 age-matched healthy control persons. The clinical characteristics of the patients are shown in Table 1. Twelve Parkinson’s disease patients had been diagnosed with impaired glucose tolerance or T2DM and HbA1c levels were maintained between 5.7–6.4% by diet control with/without medication.

Cohort 2 consisted of 145 patients with Parkinson’s disease and 45 age-matched healthy controls. Their clinical characteristics are shown in Table 1. Thirteen of the Parkinson’s disease patients had been diagnosed with impaired glucose tolerance or T2DM and their HbA1c levels were controlled between 5.9–7.0% by diet with or without medication.

### Assessment of clinical symptoms

The clinical conditions of the Parkinson’s disease patients were evaluated based on criteria established by H&Y stages^63^, UPDRS-III,MMSE, and occurrences of wearing off and dyskinesia (defined as motor fluctuations) were administered at the same time as blood sampling^27^. Although disease severity may be proportional to the “off” score, for practical and ethical reasons, the H&Y stage and UPDRS-III rating were defined in the “on state” when patients reported the effects of the last dose of medication was optimal. Patients with Parkinson’s disease were classified as shown in Table 1 and were being treated with a variety of antiparkinsonian drugs. Based on medication history, the levodopa equivalent doses were calculated according to the guideline published by Hobson *et al*.^64^.

### Sample collection

All fasting blood samples were collected at the outpatient department of Juntendo University Hospital between December 2014 and February 2015. Following an overnight fast (12–14 h), a plasma sample was obtained using 7 ml EDTA-2Na blood spits (PN7®, SRL) followed by two or three inversions. The samples were then allowed to rest for 30–60 minutes at 4 °C followed by centrifugation for 10 minutes at 2,660 × *g*. The plasma was then separated and placed in collection tubes, which were then stored in liquid nitrogen until analysis. Comprehensive metabolomics analysis was performed in February 2014 (1^st^ cohort) or February 2015 (2^nd^ cohort).

### Metabolite extraction

Metabolite extraction and metabolome analysis were conducted at Human Metabolome Technologies (HMT), Japan. For CE-TOFMS analysis, 50 μl of plasma was added to 450 μl of methanol containing internal standards (H3304–1002, HMT) on ice. The solution was then mixed thoroughly with 500 μl chloroform and 200 μl Milli-Q water and centrifuged at 2,300 × *g* for 5 min at 4 °C. The upper aqueous layer was centrifugally filtered through Millipore 5 kDa cut-off filter (UltrafreeMC-PLHCC, HMT) at 9,100 × *g* for 120 min at 4 °C to remove macromolecules. The filtrate was then centrifugally concentrated and reconstituted in 25 μl Milli-Q water prior to CE-TOFMS analysis.

For LC-TOFMS analysis, 500 μl plasma samples were added to 1,500 μl acetonitrile with 1% formic acid containing internal standard solution (H3304-1002, HMT) on ice. The solution was then mixed thoroughly and centrifuged at 2,300 × *g* for 5 min at 4 °C. The supernatant was applied to a Hybrid SPE phospholipid cartridge (55261-U, Sigma-Aldrich). The filtrate was dried by nitrogen gas and reconstituted in 200 μl of 50% isopropanol prior to LC-TOFMS analysis.

### Metabolome analysis

Metabolome analysis was conducted by CE-TOFMS and LC-TOFMS using Advanced Scan Plus (HMT) based on methods described previously^65, 66^. Briefly, CE-TOFMS analysis was performed using an Agilent CE system equipped with a 6210 TOFMS, a 1100 series binary high performance liquid chromatography pump, a G1603A CE-MS adapter kit and a G1607A CE-ESI-MS sprayer kit (all Agilent Technologies, CA, United States). The systems were controlled by Agilent G2201AA ChemStation software (Agilent Technologies) and connected by a fused silica capillary (50 μm i.d. ×80 cm) filled with commercial electrophoresis buffer (H3301-1001 and H3302-1021 for cation and anion analyses, respectively; HMT). Exact mass data were acquired over a 50–1000 *m/z* range. LC-TOFMS analysis was carried out using a 1200 series RRLC system SL and a 6230 TOFMS (both Agilent Technologies) equipped with ODS column (2 × 50 mm, 2 μm). The systems were controlled by G2201AA ChemStation software.

Data obtained by both CE-TOFMS and LC-TOFMS were processed by MasterHands (Keio University, Tsuruoka, Yamagata, Japan) to extract peak information including *m/z*, peak area, and MT for CE-TOFMS, and RT for LC-TOFMS. Signal peaks corresponding to isotopomers, adduct ions, and other product ions of known metabolites were excluded. The remaining peaks were annotated according to the HMT metabolite database based on their *m*/*z* values with the MTs or RTs. The areas of the annotated peaks were then normalized based on internal standard levels and sample volumes for relative quantification.

### Biochemical measurements

Total creatine kinase was measured using automated enzymatic techniques (Sysmex Inc., Japan). Aldolase was measured by a coupled enzyme assay (Alfresa Pharma, UK) and HbA1c was measured by boronate affinity high performance liquid chromatography, in accordance with standard protocols. NEFA was measured using an enzymatic method (NEFAzyme Kit®, Eiken, Japan).

### Statistical analysis

Plasma metabolome data were *z*-value transformed and PCA and HCA applied using PeakStat and SampleStat, respectively (HMT). Wilcoxon rank sum test was performed by R (ver. 3.2.3) (https://www.r-project.org/) for testing statistical significance between controls and patients with Parkinson’s disease in both cohorts. Steel’s test, a nonparametric, multiple-comparison test for comparing each H&Y stage (I, II, III, IV) to controls, was used. ROC analysis was carried out using JMP®12 (SAS Institute Inc.) or SAS version 9.4 (SAS Institute Inc.) and the optimal cut-off value and AUC were calculated. Pearson’s correlation coefficient was used to assess the relationships between the levels of ACs and FAs and LED or UPDRS-III scores in Parkinson’s disease using JMP®12. MANOVA was used to assess the effects of age and disease severity on LCACs using JMP®12. A P-value of less than 0.05 was considered statistically significant.

### PCA and HCA

PCA is the most common multivariate data analysis method, and is used to reduce the dimensionality of a multivariate dataset by data decomposition. This method is designed to extract the X-data matrix and maximum variation. As this modeling is done solely on the explanatory variables and without any prior information of the samples, PCA is an unsupervised and hence unbiased method. PCA was applied prior to the detailed data analysis as previously noted^67^. In this study, the procedure was repeated until the datasets were presented within three dimensions. We also visualized the observed metabolomic profile as a heat map representation, and performed hierarchical clustering analysis. The metabolite concentrations were averaged in each group, and the colours on the heat map were determined by subtracting the mean over four groups after log2 transformation. Euclidean distance was used for clustering metabolites.

## Electronic supplementary material

Supplementary information

## References

[1] Jankovic J, Poewe W (2012). Therapies in Parkinson’s disease. Current opinion in neurology.

[2] Postuma RB, Lang AE, Gagnon JF, Pelletier A, Montplaisir JY (2012). How does parkinsonism start? Prodromal parkinsonism motor changes in idiopathic REM sleep behaviour disorder. Brain: a journal of neurology.

[3] Koller W, Kase S (1986). Muscle strength testing in Parkinson’s disease. Eur Neurol.

[4] Gustafsson H, Aasly J, Strahle S, Nordstrom A, Nordstrom P (2015). Low muscle strength in late adolescence and Parkinson disease later in life. Neurology.

[5] Xu Q (2010). Physical activities and future risk of Parkinson disease. Neurology.

[6] Chen H, Zhang SM, Schwarzschild MA, Hernan MA, Ascherio A (2005). Physical activity and the risk of Parkinson disease. Neurology.

[7] Thacker EL (2008). Recreational physical activity and risk of Parkinson’s disease. Movement disorders: official journal of the Movement Disorder Society.

[8] Lewitt PA (2013). 3-hydroxykynurenine and other Parkinson’s disease biomarkers discovered by metabolomic analysis. Movement disorders: official journal of the Movement Disorder Society.

[9] Hatano, T., Saiki, S., Okuzumi, A., Mohney, R. P. & Hattori, N. Identification of novel biomarkers for Parkinson’s disease by metabolomic technologies. *J Neurol Neurosurg Psychiatry*, doi:10.1136/jnnp-2014-309676 (2015). 10.1136/jnnp-2014-309676 25795009

[10] Ahmed SS, Santosh W, Kumar S, Christlet HT (2009). Metabolic profiling of Parkinson’s disease: evidence of biomarker from gene expression analysis and rapid neural network detection. J Biomed Sci.

[11] Roede JR (2013). Serum metabolomics of slow vs. rapid motor progression Parkinson’s disease: a pilot study. PloS one.

[12] Luan H (2015). LC-MS-based urinary metabolite signatures in idiopathic Parkinson’s disease. J Proteome Res.

[13] Luan H (2015). Comprehensive urinary metabolomic profiling and identification of potential noninvasive marker for idiopathic Parkinson’s disease. Sci Rep.

[14] Bolner A, Pilleri M, De Riva V, Nordera GP (2011). Plasma and urinary HPLC-ED determination of the ratio of 8-OHdG/2-dG in Parkinson’s disease. Clin Lab.

[15] Paik MJ (2010). Polyamine patterns in the cerebrospinal fluid of patients with Parkinson’s disease and multiple system atrophy. Clin Chim Acta.

[16] Charlett A (1998). Cortisol is higher in parkinsonism and associated with gait deficit. Acta Neurol Scand.

[17] Djamshidian A (2011). Salivary cortisol levels in Parkinson’s disease and its correlation to risk behaviour. J Neurol Neurosurg Psychiatry.

[18] Hartmann A, Veldhuis JD, Deuschle M, Standhardt H, Heuser I (1997). Twenty-four hour cortisol release profiles in patients with Alzheimer’s and Parkinson’s disease compared to normal controls: ultradian secretory pulsatility and diurnal variation. Neurobiology of aging.

[19] Ohman A, Forsgren L (2015). NMR metabonomics of cerebrospinal fluid distinguishes between Parkinson’s disease and controls. Neuroscience letters.

[20] Olivola E (2014). Serotonin impairment in CSF of PD patients, without an apparent clinical counterpart. PloS one.

[21] Wuolikainen A (2016). Multi-platform mass spectrometry analysis of the CSF and plasma metabolomes of rigorously matched amyotrophic lateral sclerosis, Parkinson’s disease and control subjects. Mol Biosyst.

[22] Roberts, L. D. In *Metabolomics and systems biology in human health and medicine* (ed O. A. H. Jones) 141–156 (CABI, 2014).

[23] Shearer, J. & Weljie, A. M. In *Metabolomics and systems biology in human health and medicine*. (ed O. A. H. Jones) 157–170 (CABI, 2014).

[24] Soga T (2003). Quantitative metabolome analysis using capillary electrophoresis mass spectrometry. J Proteome Res.

[25] Sato S, Soga T, Nishioka T, Tomita M (2004). Simultaneous determination of the main metabolites in rice leaves using capillary electrophoresis mass spectrometry and capillary electrophoresis diode array detection. Plant J.

[26] Kami K (2013). Metabolomic profiling of lung and prostate tumor tissues by capillary electrophoresis time-of-flight mass spectrometry. Metabolomics.

[27] Goetz CG (2008). Movement Disorder Society-sponsored revision of the Unified Parkinson’s Disease Rating Scale (MDS-UPDRS): scale presentation and clinimetric testing results. Movement disorders: official journal of the Movement Disorder Society.

[28] Marusiak J, Jaskolska A, Koszewicz M, Budrewicz S, Jaskolski A (2012). Myometry revealed medication-induced decrease in resting skeletal muscle stiffness in Parkinson’s disease patients. Clin Biomech (Bristol, Avon).

[29] Adams F (2008). Influences of levodopa on adipose tissue and skeletal muscle metabolism in patients with idiopathic Parkinson’s disease. Eur J Clin Pharmacol.

[30] Nelson, D. L. & Michael, M. C. In *Lehninger Principles of Biochemistry* Ch. 17, 667–693 (Macmillan Higher Education, 2013).

[31] Mihalik SJ (2012). Metabolomic profiling of fatty acid and amino acid metabolism in youth with obesity and type 2 diabetes: evidence for enhanced mitochondrial oxidation. Diabetes Care.

[32] Mihalik SJ (2010). Increased levels of plasma acylcarnitines in obesity and type 2 diabetes and identification of a marker of glucolipotoxicity. Obesity (Silver Spring).

[33] Newgard CB (2009). A branched-chain amino acid-related metabolic signature that differentiates obese and lean humans and contributes to insulin resistance. Cell Metab.

[34] Huffman KM (2009). Relationships between circulating metabolic intermediates and insulin action in overweight to obese, inactive men and women. Diabetes Care.

[35] Ha CY (2012). The association of specific metabolites of lipid metabolism with markers of oxidative stress, inflammation and arterial stiffness in men with newly diagnosed type 2 diabetes. Clin Endocrinol (Oxf).

[36] Mai M (2013). Serum levels of acylcarnitines are altered in prediabetic conditions. PloS one.

[37] Adams SH (2009). Plasma acylcarnitine profiles suggest incomplete long-chain fatty acid beta-oxidation and altered tricarboxylic acid cycle activity in type 2 diabetic African-American women. J Nutr.

[38] Hoppel CL, Genuth SM (1980). Carnitine metabolism in normal-weight and obese human subjects during fasting. Am J Physiol.

[39] LeWitt, P. A. & Galloway, M. P. In *Therapy of Parkinson’s disease* (ed W. C. Koller, Paluson, G.) 63–93 (Marcel Dekker Inc, 1990).

[40] listed Naw (1995). Cerebrospinal fluid homovanillic acid in the DATATOP study on Parkinson’s disease. Parkinson Study Group. Archives of neurology.

[41] Guzman M (2004). Oleoylethanolamide stimulates lipolysis by activating the nuclear receptor peroxisome proliferator-activated receptor alpha (PPAR-alpha). The Journal of biological chemistry.

[42] Melland-Smith M (2015). Disruption of sphingolipid metabolism augments ceramide-induced autophagy in preeclampsia. Autophagy.

[43] Mizushima N, Komatsu M (2011). Autophagy: renovation of cells and tissues. Cell.

[44] Frake RA, Ricketts T, Menzies FM, Rubinsztein DC (2015). Autophagy and neurodegeneration. The Journal of clinical investigation.

[45] McCoin CS, Knotts TA, Adams SH (2015). Acylcarnitines–old actors auditioning for new roles in metabolic physiology. Nat Rev Endocrinol.

[46] Wyss M, Kaddurah-Daouk R (2000). Creatine and creatinine metabolism. Physiol Rev.

[47] Rodriguez-Gutierrez R (2012). Impact of an exercise program on acylcarnitines in obesity: a prospective controlled study. J Int Soc Sports Nutr.

[48] Lehmann R (2010). Medium chain acylcarnitines dominate the metabolite pattern in humans under moderate intensity exercise and support lipid oxidation. PloS one.

[49] Thompson DK (2012). Daily Variation of Serum Acylcarnitines and Amino Acids. Metabolomics.

[50] Chen H, Zhang SM, Hernan MA, Willett WC, Ascherio A (2003). Weight loss in Parkinson’s disease. Annals of neurology.

[51] Lorenzetti D (2004). The neurological mutant quaking(viable) is Parkin deficient. Mamm Genome.

[52] Krige D, Carroll MT, Cooper JM, Marsden CD, Schapira AH (1992). Platelet mitochondrial function in Parkinson’s disease. The Royal Kings and Queens Parkinson Disease Research Group. Annals of neurology.

[53] Yoshino H, Nakagawa-Hattori Y, Kondo T, Mizuno Y (1992). Mitochondrial complex I and II activities of lymphocytes and platelets in Parkinson’s disease. J Neural Transm Park Dis Dement Sect.

[54] McGarry JD, Brown NF (1997). The mitochondrial carnitine palmitoyltransferase system. From concept to molecular analysis. Eur J Biochem.

[55] Schlaepfer IR (2014). Lipid catabolism via CPT1 as a therapeutic target for prostate cancer. Mol Cancer Ther.

[56] Saiki S, Sato S, Hattori N (2012). Molecular pathogenesis of Parkinson’s disease: update. J Neurol Neurosurg Psychiatry.

[57] Blin O (1994). Mitochondrial respiratory failure in skeletal muscle from patients with Parkinson’s disease and multiple system atrophy. J Neurol Sci.

[58] Cardellach F (1993). Mitochondrial respiratory chain activity in skeletal muscle from patients with Parkinson’s disease. Neurology.

[59] Funayama M (2015). CHCHD2 mutations in autosomal dominant late-onset Parkinson’s disease: a genome-wide linkage and sequencing study. The Lancet. Neurology.

[60] Chace DH, DiPerna JC, Kalas TA, Johnson RW, Naylor EW (2001). Rapid diagnosis of methylmalonic and propionic acidemias: quantitative tandem mass spectrometric analysis of propionylcarnitine in filter-paper blood specimens obtained from newborns. Clin Chem.

[61] Jankovic J (1990). Variable expression of Parkinson’s disease: a base-line analysis of the DATATOP cohort. The Parkinson Study Group. Neurology.

[62] Postuma RB (2015). MDS clinical diagnostic criteria for Parkinson’s disease. Movement disorders: official journal of the Movement Disorder Society.

[63] Hoehn MM, Yahr MD (2001). Parkinsonism: onset, progression, and mortality. 1967. Neurology.

[64] Hobson DE (2002). Excessive daytime sleepiness and sudden-onset sleep in Parkinson disease: a survey by the Canadian Movement Disorders Group. Jama.

[65] Ohashi Y (2008). Depiction of metabolome changes in histidine-starved Escherichia coli by CE-TOFMS. Mol Biosyst.

[66] Ooga T (2011). Metabolomic anatomy of an animal model revealing homeostatic imbalances in dyslipidaemia. Mol Biosyst.

[67] Sugimoto M, Kawakami M, Robert M, Soga T, Tomita M (2012). Bioinformatics Tools for Mass Spectroscopy-Based Metabolomic Data Processing and Analysis. Curr Bioinform.

