# Supplementary material for: Decreased long-chain acylcarnitines from insufficient β-oxidation as potential early diagnostic markers for Parkinson’s disease
Source: Sci Rep. 2017 Aug 4;7:7328. doi: 10.1038/s41598-017-06767-y (PMC5544708; doi:10.1038/s41598-017-06767-y)
Supplement: Supplementary file 1 — Supplementary information [file 41598_2017_6767_MOESM1_ESM.pdf]

Supplementary information of "Decreased long-chain acylcarnitines from insufficient β-oxidation as potential early diagnostic markers for Parkinson's disease"

Shinji Saiki,[1] Taku Hatano[1], Motoki Fujimaki[1], Kei-Ichi Ishikawa[1], Akio Mori[1], Yutaka Oji[1], Ayami Okuzumi[1], Takeshi Fukuhara[1], Takahiro Koinuma[1], Yoko Imamichi[1], Miho Nagumo[1], Norihiko Furuya[1,2] Shuko Nojiri[3], Taku Amo[4], Kazuo Yamashiro[1] and Nobutaka Hattori[1,2,*]

1. Department of Neurology, Juntendo University School of Medicine, Bunkyo, Tokyo, 113-8421, Japan

2. Department of Research and Therapeutics for Movement Disorders, Juntendo University School of Medicine, Bunkyo, Tokyo, 113-8421, Japan

3. Clinical Research Center, Juntendo University, Bunkyo, Tokyo, 113-8421, Japan

4. Department of Applied Chemistry, National Defense Academy, Yokosuka, Kanagawa, 239-8686, Japan

Supplementary Figure Legends

Supplementary Figure 1 – Additional principal component analysis of plasma metabolites from 32 controls and 32 patients with Parkinson's disease arranged by PC1 and PC3.

A, C, E, G, H PCA from 32 controls and 32 patients with Parkinson's disease randomly selected from the first cohort.

B, D, F, H, J Top 15 factor loading in PC3 listed according to statistical significance.

Abbreviations: AC = acylcarnitine; CTR= control; FA = fatty acid; N-MPEA = N-methylphenylethanolamine; PD = Parkinson's disease

Supplementary Figure 2 – Each long-chain acylcarnitine ratio of Hoehn and Yahr stages II–IV to that in Hoehn and Yahr I in Parkinson's disease with or without motor fluctuations

A, D Statistical analysis was performed using Wilcoxon's test for each long-chain acylcarnitine in each Hoehn and Yahr stage in Parkinson's disease with or without motor fluctuations. No apparent trends commonly observed in both cohorts were detected.

Supplementary Figure 3 – Multiple comparisons of the metabolites associated with skeletal muscle in both cohorts.

A, B, C Statistical analysis was performed using Steel's test for skeletal muscle-associated metabolites and enzymes. It should be mentioned that no changes of carnitine levels were noted in any stage of the disease.

D No significant differences of HbA1c levels among H&Y stages were detected by Steel's test and ANOVA.

Abbreviations: CK = creatine kinase; H&Y = Hoehn and Yahr stage; Hb A1c = haemoglobin A1c; Ctrl = control

Error bars, S.D.; *P<0.05; **P<0.01 (Steel's test), NS = not significant

Supplementary Figure 4 – Levels of 3-methoxytyrosine and homovanillic acid were correlated with levodopa equivalent dose.

A, B Levels of 3-methoxytyrosine in Parkinson's disease in the first and second cohorts correlated with levodopa equivalent dose. Statistical analysis was performed using ANOVA.

C 3-Methoxytyrosine levels were much lower in all cases of normal controls as well as *de novo* Parkinson's disease.

D, E Levels of homovanillic acid in Parkinson's disease in the first and second cohorts correlated with levodopa equivalent dose. Statistical analysis was performed using ANOVA.

F Homovanillic acid levels of *de novo* Parkinson's disease were mildly decreased compared with those in normal controls, suggesting that no homovanillic acid was derived from orally administered levodopa.

Abbreviations: Ctrl = control; PD = Parkinson's disease

Error bars, S.D.; **P<0.01; ***P<0.001 (Steel-Dwass test)

Supplementary Figure 1

A

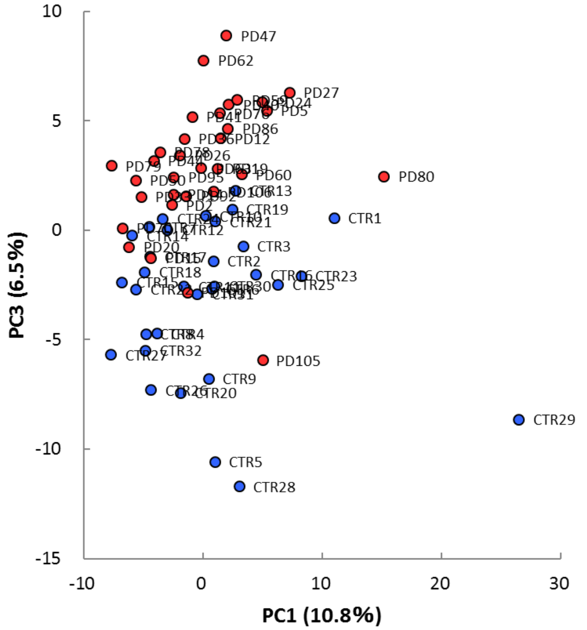

B

Factor loading in PCA (PC3)

1. AC(18:2): -0.164
2. AC(16:0): -0.160
3. AC(18:1): -0.158
4. AC(14:0): -0.147
5. AC(12:0): -0.144
6. AC(18:0): -0.134
7. AC(16:1): -0.134
8. N-MPEA: -0.128
9. AC(14:2): -0.127
10. Pyruvic acid: -0.126
11. AC(20:1): -0.120
12. Taurochenodeoxycholic acid: -0.118
13. Glycochenodeoxycholic acid: -0.115
14. Glycoursodeoxycholic acid: -0.115
15. AC(13:1): -0.115

C

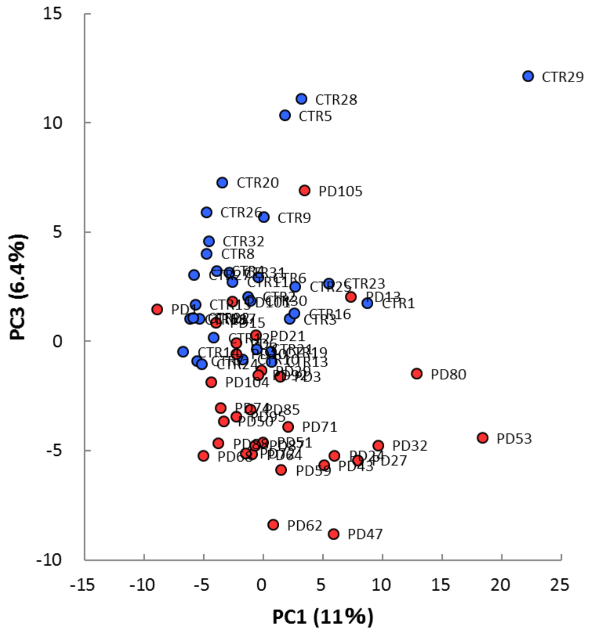

D

Factor loading in PCA (PC3)

1. AC(18:1): 0.169
2. AC(14:0): 0.168
3. AC(18:2): 0.166
4. AC(16:0): 0.163
5. AC(12:0): 0.162
6. N-MPEA: 0.156
7. AC(16:1): 0.155
8. AC(14:2): 0.142
9. AC(18:0): 0.137
10. AC(12:1): 0.135
11. Glycoursodeoxycholic acid: 0.123
12. AC(14:1): 0.119
13. AC(14:3): 0.117
14. AC(20:1): 0.115
15. AC(16:2): 0.114

Supplementary Figure 1 (continued)

E

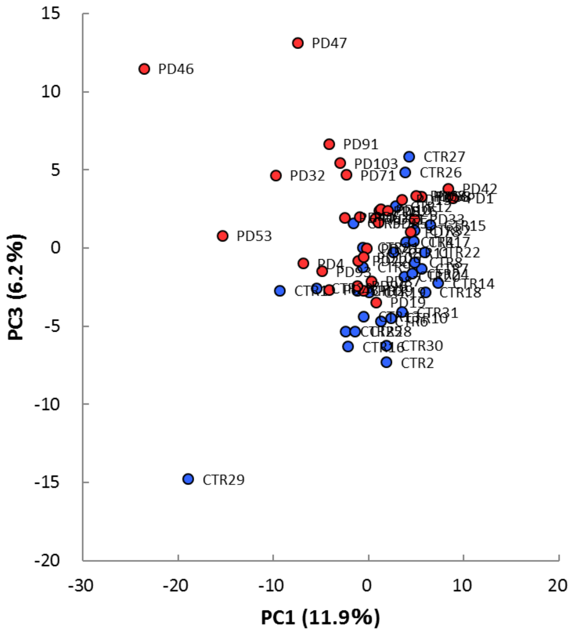

F

Factor loading in PCA (PC3)

1. AC(16:2): -0.128
2. AC(16:1): -0.118
3. AC(12:0): -0.115
4. AC(12:1): -0.114
5. AC(14:1): -0.108
6. AC(14:0): -0.108
7. AC(18:1): -0.104
8. FA(19:2): -0.100
9. FA(22:2): -0.100
10. FA(22:3): -0.100
11. FA(16:3): -0.100
12. AC(16:0): -0.0993
13. FA(24:4): -0.0990
14. AC(14:2): -0.0923
15. N-MPEA: -0.0916

G

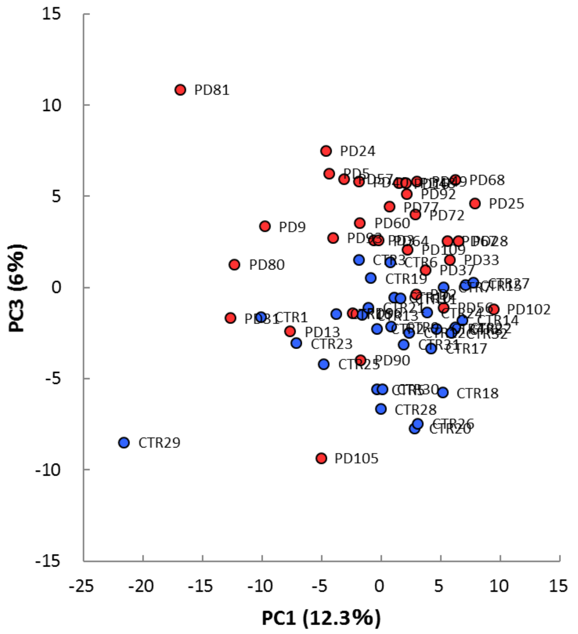

H

Factor loading in PCA (PC3)

1. AC(16:0): -0.177
2. AC(18:1): -0.164
3. AC(12:0): -0.162
4. AC(14:0): -0.160
5. AC(16:1): -0.154
6. AC(18:2): -0.150
7. AC(12:1): -0.142
8. AC(18:0): -0.141
9. AC(13:1): -0.140
10. AC(14:2): -0.136
11. AC(20:1): -0.129
12. AC(14:3): -0.127
13. AC(16:2): -0.123
14. AC(14:1): -0.119
15. N-MPEA: -0.118

Supplementary Figure 1 (continued)

I

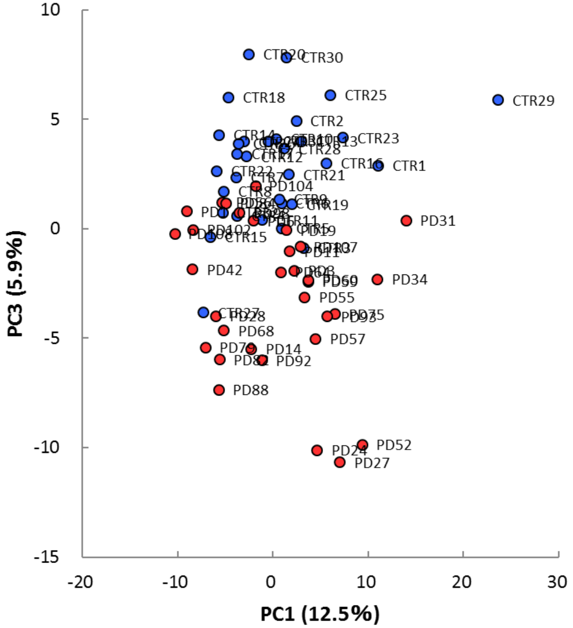

J

Factor loading in PCA (PC3)

1. AC(16:0) : 7.8E-02
2. AC(12:0) : 7.8E-02
3. AC(14:0) : 7.6E-02
4. AC(18:1) : 7.6E-02
5. AC(18:0) : 7.6E-02
6. AC(14:2) : 7.6E-02
7. AC(16:1) : 7.6E-02
8. AC(12:1) : 7.6E-02
9. AC(18:2) : 7.5E-02
10. AC(20:1) : 7.5E-02
11. AC(14:3) : 7.5E-02
12. AC(16:2) : 7.5E-02
13. N-MPEA : 7.5E-02
14. AC(14:1) : 7.4E-02
15. AC(13:1) : 7.4E-02

Supplementary Figure 2

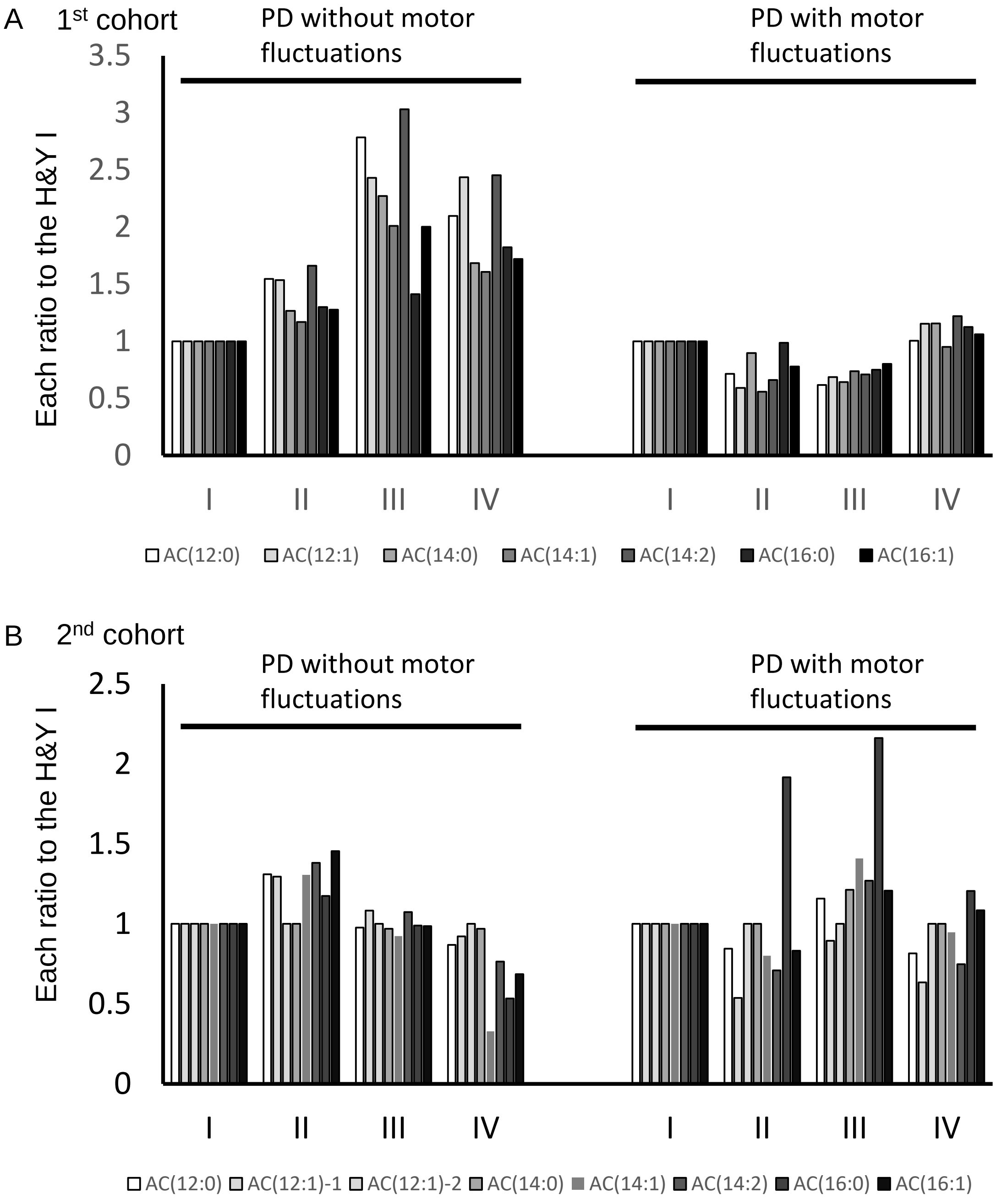

Supplementary Figure 3

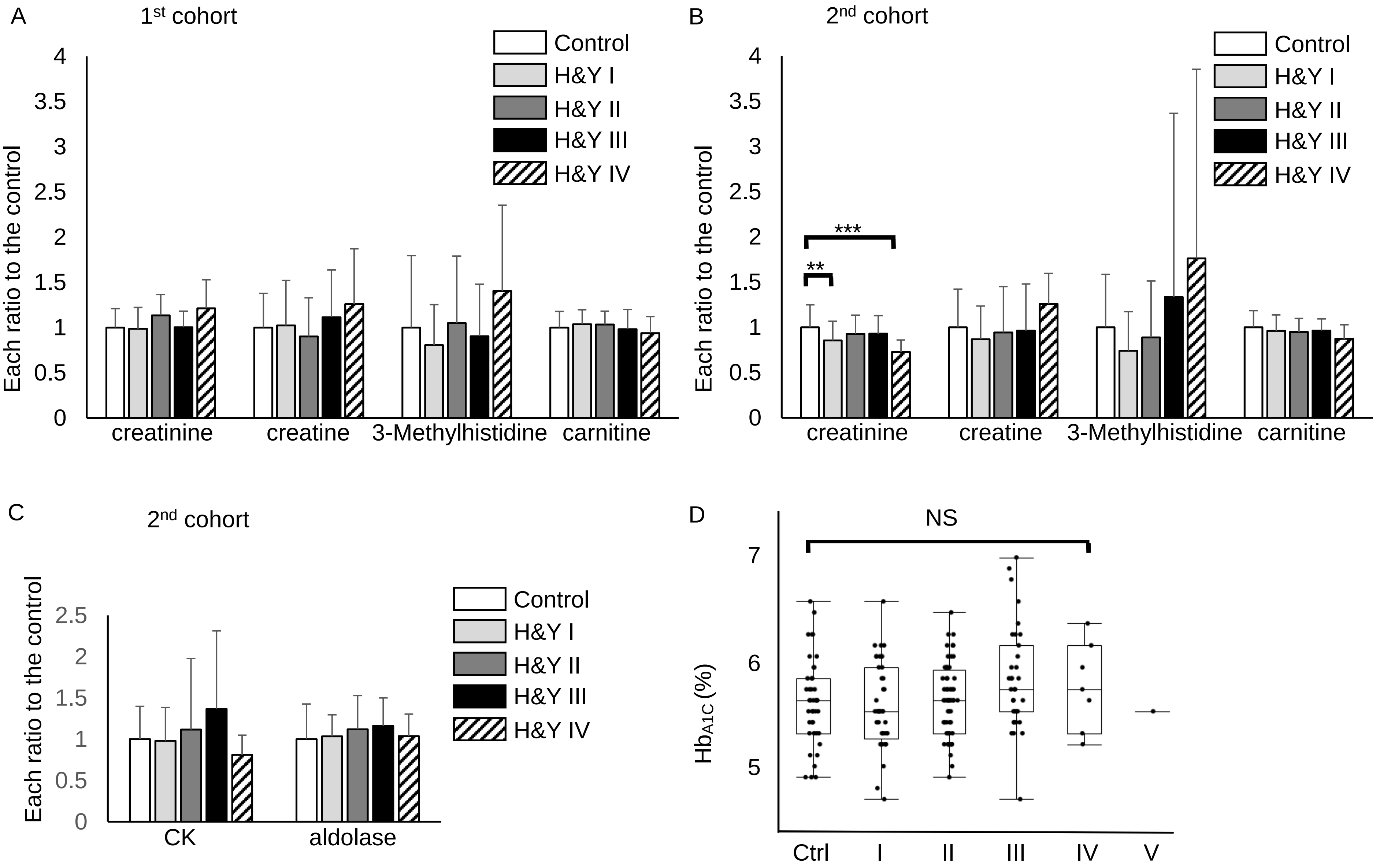

Supplementary Figure 4

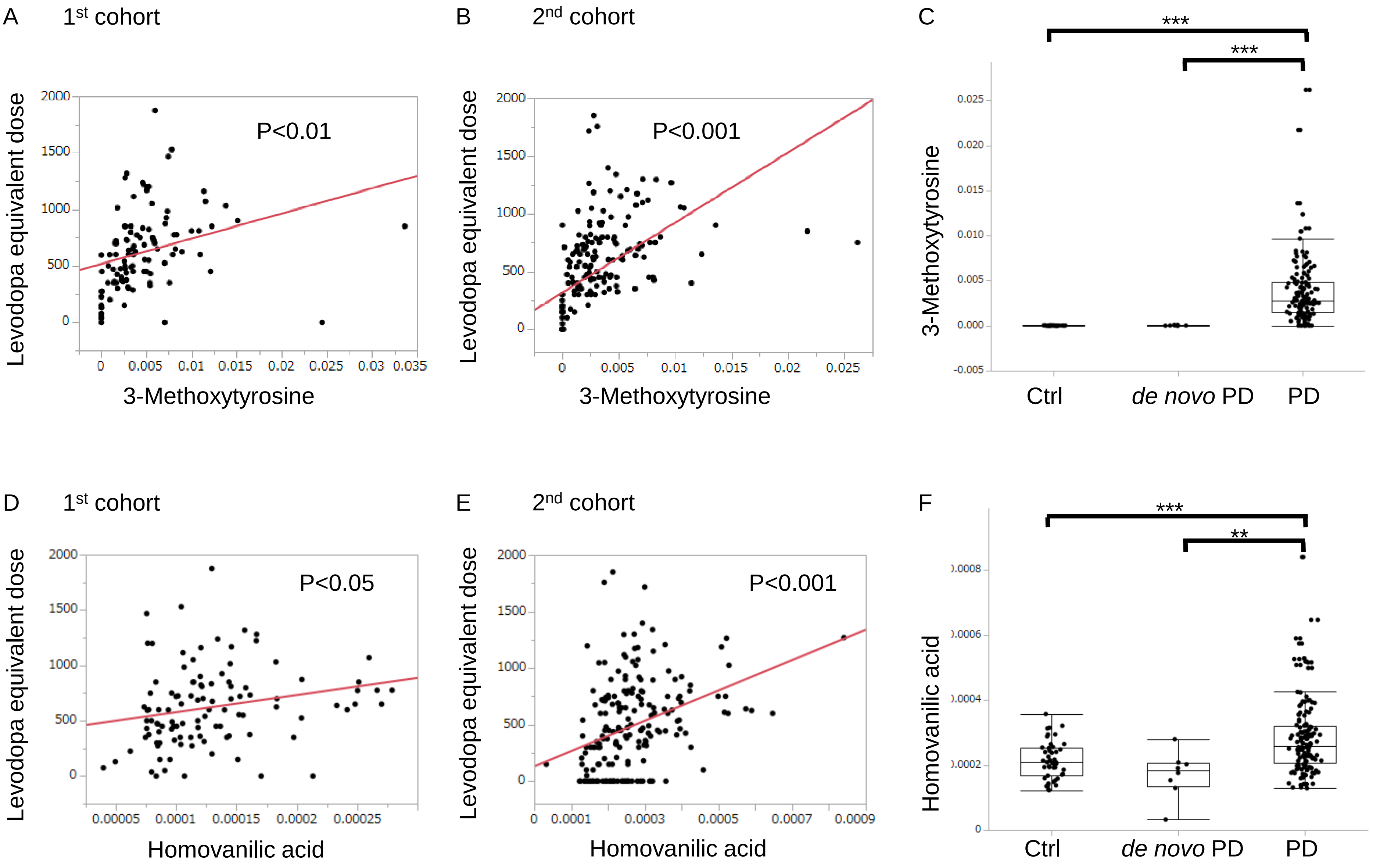

Supplementary Table S1. Multiple comparisons of clinical parameters according to H&Y stage in both cohorts

	Control		H&Y I		H&Y II		H&Y III		H&Y IV		H&Y V		
	**Mean**	***P*-value[a]**	**Mean**	***P*-value[a]**	**Mean**	***P*-value[a]**	**Mean**	***P*-value[a]**	**Mean**	***P*-value[a]**	**Mean**	***P*-value[a]**	***P*-value[b]**
**1st cohort**	**N=32**		**N=26**		**N=52**		**N=21**		**N=9**		**N=1**		
**Age [years]**	62.9	-	63.1	*1.000*	67.4	*0.372*	71.0	*0.0801*	69.0	*0.536*	67.0	-	*0.0492*
**BMI [kg/m²]**	24.1	-	22.3	*0.243*	22.3	*0.275*	21.1	*0.0264*	20.8	*0.143*	17.3	-	*0.008*
**disease duration**	-	-	3.67	-	5.89	-	7.76	-	14.6	-	10.0	-	*<0.0001*
**UPDRS-III**	-	-	5.92	-	13.0	-	17.2	-	27.7	-	34.0	-	*<0.0001*
**LED**	-	-	383 (220)	-	590 (319)	-	744 (281)	-	891 (433)	-	1650	-	*<0.0001*
**MMSE**	-	-	28.7	-	28.5	-	28.6	-	28.1	-	29.0	-	*0.579*
**2nd cohort**	**N=45**		**N=41**		**N=60**		**N=35**		**N=8**		**N=1**		
**Age [years]**	63.8	-	63.6	*0.991*	68.0	*0.898*	70.2	*0.378*	74.5	*0.220*	70.0	-	*0.0053*
**BMI [kg/m²]**	23.2	-	22.4	*0.441*	22.4	*0.510*	22.7 (4.3)	*0.975*	21.6	*0.208*	21.0	-	*0.549*
**disease duration**	-	-	4.27	-	6.54	-	9.71	-	11.6	-	21.0	-	*<0.0001*
**UPDRS-III**	-	-	7.24	-	13.6	-	22.5	-	27.1	-	31.0	-	*<0.0001*
**LED**	-	-	439 (261)	-	582 (319)	-	817 (338)	-	881 (297)	-	1050.0	-	*<0.0001*
**MMSE**	28.9	-	28.9	-	28.5 (1.6)	-	28.0	-	28.1	-	28.0	-	*0.204*

Each parameter was assessed by Steel's test and one-way ANOVA.

Abbreviations: SD = standard deviation; BMI = body mass index; H&Y stage = Hoehn and Yahr stage; UPDRS-III = Unified Parkinson's Disease Rating Scale motor section; LED = levodopa equivalent dose; MMSE = Mini Mental State Examination

[a]*P*-value obtained by Steel's test, comparing with controls. [b]*P*-value obtained by analysis of covariance among H&Y stages I-IV.

Supplementary Table S2. Multiple comparisons of short- to long-chain acylcarnitines in each cohort, corresponding to Main Figure 3.

compound name	H&Y I/control		H&Y II/control		H&Y III/control		H&Y IV/control		*P*-value[b]
	**Mean**	***P*-value[a]**	**Mean**	***P*-value[a]**	**Mean**	***P*-value[a]**	**Mean**	***P*-value[a]**	
**1st cohort**	**N=26**		**N=52**		**N=21**		**N=9**		
**Carnitine**	1.04	*0.821*	1.03	*0.823*	0.981	*0.979*	0.939	*0.821*	*0.157*
**AC(2:0)**	1.05	*0.981*	1.03	*0.941*	1.04	*0.997*	1.00	*1.00*	*0.962*
**AC(4:0)**	1.08	*0.945*	1.37	*0.0355*	1.10	*0.978*	1.33	*0.538*	*0.107*
**AC(8:0)**	1.01	*0.994*	1.04	*0.989*	1.23	*0.996*	1.26	*0.607*	*0.602*
**AC(12:0)**	0.445	*<0.0001*	0.479	*<0.0001*	0.588	*0.0008*	0.662	*0.493*	*<0.0001*
**AC(12:1)**	0.492	*0.0004*	0.487	*<0.0001*	0.635	*0.0063*	0.852	*0.993*	*0.0001*
**AC(13:1)**	0.452	*<0.0001*	0.640	*0.0002*	0.581	*0.0023*	0.909	*0.561*	*<0.0001*
**AC(14:0)**	0.472	*<0.0001*	0.515	*<0.0001*	0.584	*0.0015*	0.678	*0.356*	*<0.0001*
**AC(14:1)**	0.674	*0.2*	0.563	*0.019*	0.819	*0.328*	0.858	*1.00*	*0.0762*
**AC(14:2)**	0.525	*0.0001*	0.566	*0.0004*	0.772	*0.014*	0.930	*1.00*	*0.0002*
**AC(16:0)**	0.485	*<0.0001*	0.561	*<0.0001*	0.497	*0.0001*	0.724	*0.347*	*<0.0001*
**AC(16:1)**	0.530	*0.0002*	0.544	*<0.0001*	0.669	*0.003*	0.745	*0.584*	*<0.0001*
**AC(18:0)**	0.497	*<0.0001*	0.654	*0.0012*	0.564	*0.0007*	0.667	*0.276*	*0.0001*
**AC(18:1)**	0.510	*<0.0001*	0.575	*<0.0001*	0.548	*0.0008*	0.731	*0.956*	*<0.0001*
**AC(20:1)**	0.604	*0.0114*	0.704	*0.0197*	0.737	*0.154*	0.796	*0.807*	*0.0087*
**2nd cohort**	**N=41**		**N=60**		**N=35**		**N=8**		
**Carnitine**	0.961	*0.560*	0.947	*0.296*	0.963	*0.836*	0.873	*0.222*	*0.2229*
**AC(2:0)**	0.921	*0.698*	0.946	*0.787*	0.949	*0.895*	0.943	*0.941*	*0.977*
**AC(4:0)**	0.817	*0.149*	0.983	*0.881*	1.02	*0.994*	0.835	*0.915*	*0.0641*
**AC(8:0)**	0.807	*0.144*	1.04	*1.00*	1.05	*1.00*	0.653	*0.129*	*0.0455*
**AC(12:0)**	0.542	*0.0092*	0.685	*0.237*	0.816	*0.862*	0.626	*0.718*	*0.169*
**AC(12:1)-1**	0.671	*0.0156*	0.743	*0.233*	0.885	*0.808*	0.781	*0.915*	*0.232*
**AC(12:1)-2**	0.571	*0.0005*	0.638	*0.0073*	0.690	*0.135*	0.623	*0.354*	*0.491*
**AC(13:1)**	0.791	*0.249*	0.832	*0.649*	0.971	*1.00*	0.772	*0.904*	*0.367*
**AC(14:0)**	0.646	*0.0189*	0.761	*0.293*	0.913	*0.923*	0.771	*0.968*	*0.299*
**AC(14:1)**	0.607	*0.0144*	0.678	*0.199*	0.91	*0.882*	0.710	*0.857*	*0.269*
**AC(14:2)**	0.62	*0.0136*	0.718	*0.269*	0.89	*0.882*	0.714	*0.718*	*0.235*
**AC(16:0)**	0.664	*0.0310*	0.795	*0.286*	0.836	*0.784*	0.769	*0.815*	*0.538*
**AC(16:1)**	0.719	*0.0165*	0.820	*0.431*	0.933	*0.946*	0.881	*1.00*	*0.208*
**AC(18:0)**	0.701	*0.0530*	0.849	*0.654*	0.899	*0.959*	0.867	*0.994*	*0.208*
**AC(18:1)**	0.704	*0.0951*	0.812	*0.700*	0.882	*0.994*	0.876	*0.992*	*0.352*
**AC(20:1)**	0.837	*0.344*	0.953	*1.00*	1.06	*0.985*	0.970	*1.00*	*0.794*

Abbreviations: H&Y = Hoehn and Yahr stage; AC = acylcarnitine; NS = not significant

[a]*P*-value obtained using Steel's test, comparing with controls.

[b]*P*-value obtained by Kruskal -Wallis test, comparing H&Y stages I-IV.

Supplementary Table S3. Correlation analysis between long-chain acylcarnitines and age or BMI.

	**Age**				**Body mass index**			
	**1st cohort**		**2nd cohort**		**1st cohort**		**2nd cohort**	
**Compound name**	**r[a]**	***P*-value[b]**	**r[a]**	***P*-value[b]**	**r[a]**	***P*-value[b]**	**r[a]**	***P*-value[b]**
**AC(12:0)**	0.203	*0.0346*	0.133	*0.110*	-0.0512	0.608	0.0276	*0.742*
**AC(12:1)**	0.230	*0.0160*	-	-	-0.112	0.254	-	-
**AC(12:1)-1**	-	-	0.186	*0.0250*	-	-	-0.0170	*0.839*
**AC(12:1)-2**	-	-	0.00	-	-	-	0.00	-
**AC(14:0)**	0.219	*0.0221*	0.129	*0.121*	-0.0849	0.388	0.0272	*0.746*
**AC(14:1)**	0.141	*0.144*	0.167	*0.0452*	-0.102	0.297	-0.0394	*0.638*
**AC(14:2)**	0.226	*0.0182*	0.154	*0.0648*	-0.855	0.385	-0.0452	*0.589*
**AC(16:0)**	0.220	*0.0215*	0.106	*0.204*	-0.123	0.207	0.0680	*0.417*
**AC(16:1)**	0.263	*0.00570*	0.266	*0.00120*	-0.132	0.177	-0.0435	*0.603*

Abbreviations: BMI = body mass index; AC = acylcarnitine; r = correlation coefficient

[a, b] Correlation coefficient and *P*-value obtained by the analysis of covariance.

Supplementary Table S4. Correlation analysis with MANOVA between long-chain acylcatnitines and age

	Age (normalized by H&Y)				Age (normalized by UPDRS-III)			
	1st cohort		2nd cohort		1st cohort		2nd cohort	
**Compound name**	***F*-value**[a]	***P*-value**[b]	***F*-value**[a]	***P*-value**[b]	***F*-value**[a]	***P*-value**[b]	***F*-value**[a]	***P*-value**[b]
**AC(12:0)**	0.0735	*0.394*	0.0371	*0.401*	0.0461	*0.0919*	0.0184	*0.274*
**AC(12:1)**	0.118	*0.116*		-	0.0560	*0.0548*	-	-
**AC(12:1)-1**	-	-	0.0554	*0.181*		-	0.0359	*0.0818*
**AC(12:1)-2**	-	-	0.00	*1.00*		-	0.00	*1.00*
**AC(14:0)**	0.0779	*0.354*	0.0526	*0.206*	0.0546	*0.0599*	0.0299	*0.124*
**AC(14:1)**	0.0612	*0.523*	0.0601	*0.146*	0.0205	*0.341*	0.0336	*0.0958*
**AC(14:2)**	0.115	*0.127*	0.0608	*0.141*	0.0556	*0.0570*	0.0294	*0.127*
**AC(16:0)**	0.107	*0.160*	0.0288	*0.552*	0.0530	*0.0660*	0.0138	*0.378*
**AC(16:1)**	0.115	*0.126*	0.0946	*0.0263*	0.0747	*0.0220*	0.0795	*0.00440*

Abbreviations: MANOVA = multiple analysis of variance; UPDRS-III = Unified Parkinson's Disease Rating Scale motor section; AC = acylcarnitine

[a, b] *F*-value and *P*-value obtained by the multiple analysis of covariance.

Supplementary Table S5. Correlation analysis between long-chain acylcarnitines and UPDRS-III or levodopa equivalent dose.

	**UPDRS-III**			
	**1st cohort**		**2nd cohort**	
**Compound name**	**r**[a]	***P*-value**[b]	**r**[a]	***P*-value**[b]
**AC(12:0)**	-0.0207	*0.831*	0.0711	*0.395*
**AC(12:1)**	-0.150	*0.523*	-	-
**AC(12:1)-1**	-	-	0.0588	*0.482*
**AC(12:1)-2**	-	-	0.00950	*0.910*
**AC(14:0)**	0.0140	*0.885*	0.129	*0.122*
**AC(14:1)**	0.0347	*0.720*	0.138	*0.0991*
**AC(14:2)**	0.0618	*0.720*	0.122	*0.142*
**AC(16:0)**	-0.0196	*0.840*	0.0838	*0.316*
**AC(16:1)**	0.0109	*0.911*	0.131	*0.117*

Abbreviations: UPDRS-III = Unified Parkinson's Disease Rating Scale motor section; AC = acylcarnitine; r = correlation coefficient

[a, b]Correlation coefficient and *P*-value obtained by analysis of covariance.

Supplementary Table S6. Correlation analysis between long-chain acylcarnitines and levodopa dose or levodopa equivalent dose.

	**Levodopa dose**				**Levodopa equivalent dose**			
	**1st cohort**		**2nd cohort**		**1st cohort**		**2nd cohort**	
**Compound name**	**r[a]**	***P*-value[b]**	**r[a]**	***P*-value[b]**	**r[a]**	***P*-value[b]**	**r[a]**	***P*-value[b]**
**AC(12:0)**	-0.112	*0.215*	0.0814	*0.330*	0.0621	*0.521*	0.107	*0.201*
**AC(12:1)**	-0.163	*0.0910*	-	-	0.0864	*0.372*	-	-
**AC(12:1)-1**	-	-	0.0910	*0.276*	-	-	0.0515	*0.538*
**AC(12:1)-2**	-	-	0.00	-	-	-	0.0598	*0.475*
**AC(14:0)**	-0.162	*0.0922*	0.228	*0.00580*	0.106	*0.272*	0.145	*0.0817*
**AC(14:1)**	-0.182	*0.0588*	0.158	*0.0580*	0.0347	*0.712*	0.101	*0.229*
**AC(14:2)**	-0.146	*0.129*	0.111	*0.182*	0.107	*0.268*	0.0729	*0.383*
**AC(16:0)**	-0.115	*0.233*	0.167	*0.0451*	0.131	*0.174*	0.121	*0.149*
**AC(16:1)**	-0.210	*0.0284*	0.190	*0.0218*	0.106	*0.275*	0.110	*0.187*

Abbreviations: UPDRS-III = Unified Parkinson’s Disease Rating Scale motor section; AC = acylcarnitine; r = correlation coefficient

[a, b]Correlation coefficient and *P*-value obtained by analysis of covariance.

Supplementary Table S7. Characteristics of controls, *de novo* PD, and PD with medication in the 2nd cohort.

	Control	*de novo* PD	PD with medication	*P*-value[a]	*P*-value[c]		
					Control VS *de novo* PD	Control VS PD with medication	*de novo* PD VS PD with medication
**Number**	45	8	137	-	-	-	-
**Gender (Male:Female)**	23:22	4:4	66:71	*0.942*[b]	-	-	-
**Age [years], Mean (SD)**	63.8 (15.3)	64.6 (11.2)	67.6 (10.1)	-	*0.996*	*0.648*	*0.780*
**Duration [years], Mean (SD)**	-	1.50 (0.756)	7.37 (5.60)	*<0.0001*	-	-	-
**H&Y stage, Mean (SD)**	-	1.63 (0.916)	2.12 (0.892)	*0.121*	-	-	-
**UPDRS III, Mean (SD)**	-	12.5 (10.5)	15.0 (9.82)	*0.384*	-	-	-
**MMSE, Mean (SD)**	28.9 (2.09)	29.0 (1.69)	28.4 (1.76)	-	*0.877*	*0.0194*	*0.488*
**BMI [kg/m2]**	23.2 (3.51)	22.4 (2.54)	22.4 (3.34)	-	*0.980*	*0.745*	*0.223*

Abbreviation: PD = Parkinson's disease; H&Y stage = Hoehn and Yahr stage; UPDRS-III = Unified Parkinson's Disease Rating Scale motor section; MMSE = Mini Mental State Examination; BMI = Body Mass Index

[a]*P*-value obtained by analysis of covariance comparing *de novo* PD and PD with medication.

[b]*P*-value obtained by chi-squared test.

[c]*P*-value obtained by the Steel-Dwass test.

Supplementary Table S8. Lists of acylcarnitines and fatty acids in *de novo* PD and PD with medication.

Compound name	*de novo* PD/Control		PD with medication/Control	
	**Mean**	***P*-value[a]**	**Mean**	***P*-value[a]**
Carnitine	0.995	*0.997*	0.946	*0.072*
AC(2:0), O-Acetylcarnitine	1.03	*0.616*	0.935	*0.255*
AC(4:0), Butyrylcarnitine	0.898	*0.841*	1.16	*0.149*
AC(4:0), Isobutyrylcarnitine	0.795	*0.532*	0.943	*0.444*
AC(8:0), Octanoylcarnitine	0.84	*0.616*	0.963	*0.554*
AC(12:0)	0.411	*0.0366*	0.690	*0.0463*
AC(12:1)-1	0.421	*0.313*	0.782	*0.573*
AC(12:1)-2	0.445	*0.011*	0.643	*0.0007*
AC(13:1)	0.694	*0.452*	0.863	*0.304*
AC(14:0)	0.497	*0.0366*	0.782	*0.0783*
AC(14:1)	0.503	*0.416*	0.733	*0.0537*
AC(14:2)	0.534	*0.0966*	0.747	*0.0519*
AC(15:0)-1	0.228	*0.582*	0.296	*<0.0001*
AC(15:0)-2	0.0926	*0.0913*	0.244	*<0.0001*
AC(16:0), Palmitoylcarnitine	0.567	*0.0966*	0.779	*0.0568*
AC(16:1)	0.588	*0.0768*	0.838	*0.117*
AC(18:0)	0.664	*0.209*	0.829	*0.174*
AC(18:1)	0.595	*0.190*	0.815	*0.179*
AC(18:2)-1	0.703	*0.516*	0.842	*0.272*
AC(18:2)-2	0.698	*0.972*	1.61	*0.916*
AC(18:2)-3	0.897	*0.532*	2.436	*<0.0001*
AC(20:1)	0.929	*0.916*	0.953	*0.717*
FA(12:0)	0.882	*1.00*	1.39	*0.007*
FA(14:0), Myristic acid	0.904	*0.992*	1.24	*0.537*
FA(14:1)-1	0.748	*0.749*	1.18	*0.506*
FA(14:1)-2	0.774	*0.980*	0.976	*0.826*
FA(14:3)	0.0174	*0.0069*	1.04	*0.0121*

FA(15:0), Pentadecanoic acid	1.02	*0.421*	1.08	*0.0625*
FA(16:0), Palmitic acid	1.10	*0.437*	1.12	*0.190*
FA(16:1), Palmitoleic acid	0.879	*0.947*	1.25	*0.249*
FA(17:0), Heptadecanoic acid	1.04	*0.765*	1.04	*0.708*
FA(17:0)	1.01	*0.868*	1.01	*0.984*
FA(17:1)	0.837	*0.684*	1.02	*0.979*
FA(18:0), Stearic acid	1.10	*0.285*	1.05	*0.464*
FA(18:1), Oleic acid	1.08	*0.532*	1.15	*0.324*
FA(18:1), Ricinoleic acid	0.588	*0.005*	0.955	*0.0714*
FA(18:2), Linoleic acid	1.28	*0.199*	1.13	*0.289*
FA(18:3), Linolenic acid	1.20	*0.273*	1.23	*0.245*
FA(18:4), Stearidonic acid	0.925	*0.650*	0.924	*0.837*
FA(19:1)	0.997	*0.955*	1.00	*0.957*
FA(20:1), cis-11-Eicosenoic acid,	1.41	*0.199*	1.20	*0.217*
FA(20:2), cis-11,14-Eicosadienoic acid	1.06	*0.684*	1.05	*0.958*
FA(20:3), cis-8,11,14-Eicosatrienoic acid	0.913	*0.955*	0.956	*0.611*
FA(20:3)	0.790	*0.781*	1.066	*0.959*
FA(20:4), Arachidonic acid	1.02	*0.781*	0.956	*0.492*
FA(20:5), cis-5,8,11,14,17-Eicosapentaenoic acid	1.49	*0.392*	0.971	*0.999*
FA(22:1), Erucic acid	0.334	*1.00*	0.400	*0.335*
FA(22:4)	0.865	*0.977*	1.08	*1.00*
FA(22:5)-1	0.970	*0.927*	0.974	*0.567*
FA(22:5)-2	1.18	*0.363*	1.05	*0.982*
FA(22:6), cis-4,7,10,13,16,19-Docosahexaenoic acid	1.13	*0.437*	1.06	*0.926*
FA(24:1), Nervonic acid	1.09	*0.273*	1.05	*0.172*
FA(24:5)	0.919	*0.947*	0.959	*0.804*
NEFA	1.08	*0.933*	1.07	*0.669*

Abbreviations: PD = Parkinson's disease; AC = acylcarnitine; FA = fatty acid; NEFA = non-esterified fatty acid

[a]*P*-value obtained by Steel's test.

Supplementary Table S9. Comparison of 7 long-chain acylcarnitines in PD with/without motor fluctuations.

Comparative Analysis				
**Compound**	**PD with motor fluctuations/PD without motor fluctuations**			
	**1st cohort**		**2nd cohort**	
	**Ratio**	***P*-value[a]**	**Ratio**	***P*-value[a]**
**AC(12:0)**	0.952	*0.825*	1.11	*0.613*
**AC(12:1)**	0.867	*0.248*		
**AC(12:1)-1**	-	-	1.22	*0.211*
**AC(12:1)-2**	-	-	1.00	-
**AC(14:0)**	0.854	*0.557*	1.05	*0.552*
**AC(14:1)**	0.926	*0.439*	1.39	*0.0800*
**AC(14:2)**	0.892	*0.658*	1.17	*0.448*
**AC(16:0)**	0.943	*0.978*	1.18	*0.149*
**AC(16:1)**	0.839	*0.279*	1.51	*0.00650*

Abbreviations: PD = Parkinson's disease; AC = acylcarnitine

[a]*P*-value obtained by Wilcoxon's test between PD with and without motor fluctuations.

Supplementary Table S10. Comparison of long-chain acylcarnitines in each H&Y stage of PD with or without motor fluctuations

		PD without motor fluctuations (N)	PD with motor fluctuations (N)	Comparative Analysis																	
				PD with motor fluctuations/PD without motor fluctuations																	
				AC(12:0)		AC(12:1)		AC(12:1)-1		AC(12:1)-2		AC(14:0)		AC(14:1)		AC(14:2)		AC(16:0)		AC(16:1)	
				Ratio	*P*-value[a]	Ratio	*P*-value[a]	Ratio	*P*-value[a]	Ratio	*P*-value[a]	Ratio	*P*-value[a]	Ratio	*P*-value[a]	Ratio	*P*-value[a]	Ratio	*P*-value[a]	Ratio	*P*-value[a]
**1st cohort**	**I**	16	10	1.99	*0.848*	1.87	*0.219*	-	-	-	-	1.34	*0.886*	1.65	*0.789*	1.99	*0.763*	1.27	*0.895*	1.33	*0.859*
	**II**	26	26	0.923	*0.817*	0.719	*0.237*	-	-	-	-	0.950	*0.902*	0.786	*0.261*	0.794	*0.484*	0.967	*0.862*	0.812	*0.483*
	**III**	8	13	0.443	*0.913*	0.530	*0.364*	-	-	-	-	0.379	*0.446*	0.605	*0.561*	0.467	*0.385*	0.676	*0.971*	0.531	*0.538*
	**IV**	5	4	0.955	*1.00*	0.885	*0.793*	-	-	-	-	0.919	*0.901*	0.973	*0.893*	0.991	*0.903*	0.785	*1.00*	0.820	*0.903*
**2nd cohort**	**I**	31	10	1.29	*0.323*	-	-	1.89	*0.0748*	1.00	-	0.969	*0.61*	1.44	*0.937*	1.43	*0.229*	1.01	*0.963*	1.66	*0.191*
	**II**	33	27	0.834	*0.216*	-	-	0.780	*0.179*	1.00	-	0.968	*0.388*	0.885	*0.683*	0.731	*0.0891*	1.05	*0.858*	0.948	*0.876*
	**III**	11	24	1.53	*0.232*	-	-	1.56	*0.184*	1.00	-	1.21	*0.242*	2.20	*0.0441*	1.69	*0.173*	1.41	*0.213*	2.03	*0.0405*
	**IV**	1	7	1.21	-	-	-	1.30	-	1.00	-	1.00	-	4.17	-	1.39	-	1.02	-	2.63	-

Abbreviations: PD = Parkinson's disease; AC = acylcarnitine

[a]*P*-value obtained by Wilcoxon's test between PD with and without motor fluctuations.

Supplementary Table S11. Area under the curve of each long-chain acylcarnitine in both cohorts.

Compound name	Area under the curve value	
	1st cohort	2nd cohort
**AC(12:0)**	0.802	0.612
**AC(12:1)**	0.764	0.617
**AC(12:1)-1**	-	0.685
**AC(12:1)-2**	-	0.685
**AC(13:1)**	0.779	0.571
**AC(14:0)**	0.783	0.601
**AC(14:1)**	-	0.618
**AC(14:2)**	0.745	0.605
**AC(14:3)**	0.575	-
**AC(15:0)**	0.509	-
**AC(15:0)-1**	-	0.721
**AC(15:0)-2**	-	0.765
**AC(16:0)**	0.805	0.607
**AC(16:1)**	0.757	0.590
**AC(16:2)**	0.641	-
**AC(18:0)**	0.756	0.587
**AC(18:1)**	0.769	0.584
**AC(18:2)**	0.791	-
**AC(18:2)-1**	-	0.574
**AC(18:2)-2**	-	0.507
**AC(18:2)-3**	-	0.713
**AC(20:1)**	0.670	0.522

Abbreviation: AC = acylcarnitine

Supplementary Table S12. List of amino acids and fatty acids detected in each cohort.

Comparative Analysis

**Amino Acid**	**PD/Control**			
	**1st cohort**		**2nd cohort**	
	**Ratio**	***P*-value[a]**	**Ratio**	***P*-value[a]**
**Leu**	0.998	*0.918*	0.973	*0.254*
**Ile**	0.984	*0.949*	0.962	*0.180*
**Val**	0.980	*0.532*	0.948	*0.0759*
**Gly**	1.10	*0.0311*	1.04	*0.299*
**Arg**	1.08	*0.059*	0.953	*0.137*
**Gln**	1.06	*0.0074*	1.03	*0.232*
**Phe**	1.03	*0.516*	0.990	*0.166*
**His**	1.02	*0.832*	0.946	*0.0027*
**Pro**	0.99	*0.491*	0.994	*0.582*
**Tyr**	0.986	*0.168*	0.982	*0.319*
**Lys**	0.986	*0.626*	0.911	*0.0005*
**Ser**	0.982	*0.708*	0.951	*0.120*
**Asn**	0.970	*0.164*	0.959	*0.0403*
**Trp**	0.948	*0.318*	0.875	*<0.0001*
**Asp**	0.903	*0.129*	0.982	*0.823*
**Thr**	0.938	*0.268*	0.952	*0.127*
**Met**	0.936	*0.249*	0.935	*0.0867*
**Ala**	0.920	*0.0392*	0.921	*0.0169*
**Glu**	0.876	*0.148*	0.970	*0.806*
**FA(12:0)**	1.21	*0.39*	1.36	*0.0055*
**FA(14:0), Myristic acid**	1.14	*0.193*	1.22	*0.0351*
**FA(14:1)-1**	0.989	*0.898*	1.15	*0.375*
**FA(14:1)-2**	1.0896539	*0.958*	0.965	*0.812*
**FA(14:3)**	2.60	*<0.0001*	0.983	*0.003*
**FA(15:0), Pentadecanoic acid**	-	-	1.08	*0.0294*

**FA(16:0), Palmitic acid**	1.08	*0.389*	1.12	*0.0908*
**FA(16:1), Palmitoleic acid**	1.13	*0.291*	1.23	*0.17*
**FA(17:0)**	1.09	*0.321*	1.02	*0.838*
**FA(17:0), Heptadecanoic acid**	1.15	*0.051*	1.04	*0.443*
**FA(17:1)**	1.11	*0.348*	1.01	*0.792*
**FA(18:0), Stearic acid**	1.10	*0.138*	1.06	*0.226*
**FA(18:1), Oleic acid**	1.12	*0.259*	1.14	*0.165*
**FA(18:1), Ricinoleic acid**	-	-	0.934	*0.0184*
**FA(18:2), Linoleic acid**	1.20	*0.146*	1.14	*0.126*
**FA(18:3), Linolenic acid**	1.20	*0.223*	1.23	*0.11*
**FA(18:4), Stearidonic acid**	-	-	0.924	*0.553*
**FA(19:1)**	1.09	*0.249*	1.00	*0.835*
**FA(20:1), cis-11-Eicosenoic acid,**	1.29	*0.0418*	1.21	*0.0926*
**FA(20:2), cis-11,14-Eicosadienoic acid**	0.946	*0.293*	1.05	*0.738*
**FA(20:3)**	0.842	*0.883*	1.05	*0.754*
**FA(20:3), cis-8,11,14-Eicosatrienoic acid**	0.946	*0.99*	0.954	*0.392*
**FA(20:4), Arachidonic acid**	0.947	*0.939*	0.959	*0.36*
**FA(20:5), cis-5,8,11,14,17-Eicosapentaenoic acid**	1.40	*0.242*	0.999	*0.916*
**FA(22:4)**	0.849	*0.885*	1.07	*1.00*
**FA(22:5)**	1.13	*0.703*	-	-
**FA(22:6), cis-4,7,10,13,16,19-Docosahexaenoic acid**	1.14	*0.511*	1.07	*0.642*

Abbreviations: PD = Parkinson's Disease; FA = fatty acid

[a]*P*-value obtained by Wilcoxon's test, comparing between PD and controls.

Supplementary Table S13. Multiple comparisons of the long-chain fatty acids in each H&Y stage of both cohorts.

Comparative Analysis																		
Compound name	1st cohort									2nd cohort								
	H&Y I/Control		H&Y II/Control		H&Y III/Control		H&Y IV/Control		*P*-value[b]	H&Y I/Control		H&Y II/Control		H&Y III/Control		H&Y IV/Control		*P*-value[b]
	N=41		N=60		N=35		N=8			N=41		N=60		N=35		N=8		
	Ratio	*P*-value[a]	Ratio	*P*-value[a]	Ratio	*P*-value[a]	Ratio	*P*-value[a]		Ratio	*P*-value[a]	Ratio	*P*-value[a]	Ratio	*P*-value[a]	Ratio	*P*-value[a]	
**FA(12:0)**	1.31	*0.859*	1.19	*0.978*	0.983	*1.00*	1.35	*0.913*	*0.199*	0.958	*0.926*	1.59	*0.0275*	1.38	*0.0332*	1.67	*0.102*	*0.0099*
**FA(14:0), Myristic acid**	1.19	*0.517*	1.12	*0.734*	1.02	*1.00*	1.25	*0.938*	*0.202*	1.11	*0.913*	1.31	*0.137*	1.14	*0.51*	1.45	*0.115*	*0.1198*
**FA(14:1)-1**	-	-	-	-	-	-	-	-	-	1.01	*1.00*	1.23	*0.635*	1.00	*1.00*	1.50	*0.180*	*0.227*
**FA(14:1)-2**	-	-	-	-	-	-	-	-	-	0.984	*0.980*	0.973	*1.00*	0.926	*0.996*	0.837	*1.00*	*0.716*
**FA(14:3)**	2.73	*<0.0001*	2.42	*<0.0001*	2.97	*<0.0001*	2.52	*0.0005*	*<0.0001*	1.13	*0.0318*	1.04	*0.166*	0.754	*0.0318*	0.941	*0.701*	*0.0539*
**FA(15:0), Pentadecanoic acid**	-	-	-	-	-	-	-	-	-	1.03	*0.780*	1.10	*0.101*	1.08	*0.429*	1.13	*0.593*	*0.266*
**FA(16:0), Palmitic acid**	1.09	*0.802*	1.08	*0.801*	0.997	*1.00*	1.14	*1.00*	*0.455*	1.13	*0.751*	1.15	*0.445*	1.03	*0.877*	1.19	*0.311*	*0.377*
**FA(16:1), Palmitoleic acid**	1.18	*0.665*	1.09	*0.986*	1.08	*0.992*	1.32	*0.538*	*0.223*	1.18	*0.973*	1.30	*0.467*	1.06	*1.00*	1.58	*0.136*	*0.166*
**FA(17:0), Heptadecanoic acid**	1.15	*0.409*	1.16	*0.191*	1.11	*0.703*	1.06	*1.00*	*0.286*	1.01	*1.00*	1.06	*0.837*	0.988	*1.00*	1.13	*0.718*	*0.720*
**FA(17:0)**	1.14	*0.830*	1.10	*0.822*	1.05	*0.983*	0.936	*1.00*	*0.832*	1.02	*1.00*	1.01	*0.991*	0.978	*1.00*	1.16	*0.752*	*0.921*
**FA(17:1)**	1.12	*0.839*	1.13	*0.838*	1.02	*1.00*	1.11	*0.999*	*0.542*	0.992	*0.817*	1.07	*0.990*	0.890	*0.857*	1.14	*0.784*	*0.479*
**FA(18:0), Stearic acid**	1.12	*0.539*	1.12	*0.312*	1.05	*0.998*	0.983	*0.994*	*0.335*	1.05	*0.865*	1.06	*0.890*	1.02	*0.992*	1.21	*0.190*	*0.336*
**FA(18:1), Oleic acid**	1.15	*0.772*	1.11	*0.838*	1.07	*0.995*	1.20	*0.982*	*0.341*	1.12	*0.987*	1.19	*0.458*	1.03	*0.982*	1.36	*0.190*	*0.281*
**FA(18:1), Ricinoleic acid**	-	-	-	-	-	-	-	-	-	0.922	*0.0240*	0.959	*0.631*	0.814	*0.0563*	1.40	*0.830*	*0.0268*

**FA(18:2), Linoleic acid**	1.19	*0.653*	1.19	*0.777*	1.13	*0.888*	1.36	*0.720*	*0.380*	1.10	*0.938*	1.21	*0.522*	1.05	*0.836*	1.17	*0.370*	*0.397*
**FA(18:3), Linolenic acid**	1.15	*0.821*	1.19	*0.982*	1.10	*0.919*	1.51	*0.367*	*0.352*	1.13	*0.956*	1.33	*0.658*	1.13	*0.574*	1.32	*0.0955*	*0.258*
**FA(18:4), Stearidonic acid**	-	-	-	-	-	-	-	-	-	0.856	*1.00*	0.900	*0.997*	0.900	*0.967*	1.33	*0.385*	*0.314*
**FA(19:1)**	1.13	*0.573*	1.06	*0.833*	1.05	*0.995*	1.12	*1.00*	*0.579*	0.993	*0.932*	1.02	*0.999*	0.941	*0.882*	1.18	*0.575*	*0.533*
**FA(20:1), cis-11-Eicosenoic acid**	1.25	*0.495*	1.23	*0.367*	1.29	*0.383*	1.44	*0.783*	*0.173*	1.16	*0.801*	1.16	*0.709*	1.21	*0.759*	1.65	*0.0405*	*0.081*
**FA(20:2), cis-11,14-Eicosadienoic acid**	1.06	*0.741*	1.06	*0.771*	0.987	*0.992*	0.971	*0.989*	*0.351*	1.09	*1.00*	1.08	*0.981*	0.939	*0.994*	1.10	*0.784*	*0.702*
**FA(20:3), cis-8,11,14-Eicosatrienoic acid**	0.963	*0.987*	0.964	*1.00*	0.880	*0.927*	0.880	*0.888*	*0.470*	0.988	*0.999*	0.977	*1.00*	0.867	*1.693*	0.961	*0.370*	*0.864*
**FA(20:3)**	0.754	*0.998*	0.883	*0.984*	0.808	*0.997*	0.857	*1.00*	*0.363*	1.14	*0.953*	1.12	*0.971*	0.785	*0.771*	1.28	*1.00*	*0.221*
**FA(20:4), Arachidonic acid**	0.930	*1.00*	0.971	*0.999*	0.851	*0.933*	0.990	*1.00*	*0.673*	1.02	*0.956*	0.972	*0.855*	0.830	*0.752*	1.09	*0.941*	*0.579*
**FA(20:5), cis-5,8,11,14,17-Eicosapentaenoic acid**	1.24	*0.986*	1.53	*0.65*	0.992	*0.993*	1.59	*0.902*	*0.717*	1.10	*0.999*	0.958	*1.00*	0.889	*1.00*	1.16	*0.857*	*0.731*
**FA(22:4)**	0.863	*0.927*	0.850	*0.999*	0.797	*0.968*	0.828	*0.902*	*0.323*	1.23	*1.00*	1.05	*1.00*	0.847	*0.962*	1.28	*0.469*	*0.274*
**FA(22:5)**	1.04	*0.990*	1.18	*0.989*	0.951	*1.00*	1.17	*0.957*	*0.920*	1.19	0.909	1.01	0.932	0.953	0.952	1.09	0.990	*0.494*
**FA(22:6), cis-4,7,10,13,16,19-Docosahexaenoic acid**	1.06	*1.00*	1.20	*0.949*	1.01	*0.968*	1.05	*0.999*	*0.927*	1.10	*1.00*	1.02	*1.00*	1.06	*0.985*	1.14	*0.956*	*0.799*
**FA(24:1), Nervonic acid**	1.29	*0.471*	1.29	*0.111*	1.66	*0.0222*	1.34	*0.399*	*0.0326*	0.994	*0.978*	1.03	*0.578*	1.07	*0.482*	1.31	*0.0122*	*0.0182*
**NEFA**	-	-	-	-	-	-	-	-	-	1.06	*0.999*	1.07	*0.962*	1.05	*0.988*	1.16	*0.675*	*0.795*

Abbreviations: H&Y = Hoehn and Yahr stage; FA = fatty acid; NEFA = non-esterified fatty acid

[a]*P*-value obtained by Steel's test, comparing with controls.

[b]*P*-value obtained by Kruskal-Wallis test, comparing between H&Y stages I-IV.
